# Tilt Table Therapies for Patients with Severe Disorders of Consciousness: A Randomized, Controlled Trial

**DOI:** 10.1371/journal.pone.0143180

**Published:** 2015-12-01

**Authors:** Carmen Krewer, Marianne Luther, Eberhard Koenig, Friedemann Müller

**Affiliations:** Schoen Klinik Bad Aibling, Motor Research Department, Bad Aibling, Germany; Ludwig-Maximilian University, GERMANY

## Abstract

One major aim of the neurological rehabilitation of patients with severe disorders of consciousness (DOC) is to enhance patients’ arousal and ability to communicate. Mobilization into a standing position by means of a tilt table has been shown to improve their arousal and awareness. However, due to the frequent occurrence of syncopes on a tilt table, it is easier to accomplish verticalization using a tilt table with an integrated stepping device. The objective of this randomized controlled clinical trial was to evaluate the effectiveness of a tilt table therapy with or without an integrated stepping device on the level of consciousness. A total of 50 participants in vegetative or minimally conscious states 4 weeks to 6 month after injury were treated with verticalization during this randomized controlled trial. Interventions involved ten 1-hour sessions of the specific treatment over a 3-week period. Blinded assessors made measurements before and after the intervention period, as well as after a 3-week follow-up period. The coma recovery scale-revised (CRS-R) showed an improvement by a median of 2 points for the group receiving tilt table with integrated stepping (Erigo). The rate of recovery of the group receiving the conventional tilt table therapy significantly increased by 5 points during treatment and by an additional 2 points during the 3-week follow-up period. Changes in spasticity did not significantly differ between the two intervention groups. Compared to the conventional tilt table, the tilt table with integrated stepping device failed to have any additional benefit for DOC patients. Verticalization itself seems to be beneficial though and should be administered to patients in DOC in early rehabilitation.

***Trial Registration*:** Current Controlled Trials Ltd (www.controlled-trials.com), identifier number ISRCTN72853718

## Introduction

Many circumstances, such as traumatic brain injury, massive intracerebral bleeding, hypoxia or other forms of diffuse brain injury, may lead to severe disorders of consciousness (DOC). The consequences for patients and their families are disastrous. Severe DOC range from coma via vegetative state (VS) [1]—also referred to as unresponsive wakefulness syndrome [2], a condition in which the patient is awake but shows no behavioral evidence of conscious awareness—to minimally conscious state (MCS), characterized by one or more minimal but definite behavioral signs of consciousness [3,4]. In these cases the primary aims of neurological rehabilitation are: prevention of secondary medical complications and restoration of cognitive-behavioural functions [5].

Only limited evidence is available for treatments that have been suggested to enhance arousal and the ability to communicate, and therewith to accelerate recovery after prolonged DOC [5–9]. Drugs are commonly used to enhance arousal and promote behavioral responsiveness, but only in 2012 was the first drug amantadine HCl proven effective in a placebo-controlled trial [10]. Besides a pharmacological treatment, non-invasive brain stimulation, like transcranial direct current stimulation, might be a promising therapy option to improve signs of consciousness in patients with DOC [11]. Sensory stimulation programs (sensory stimulation via auditory, tactile, olfactory and visual inputs) were also administered to these patients. A meta-analysis published in 2002 found no reliable evidence proving the efficacy of these programs [7]. A recently published study of Urbenjaphol et al. showed that patients receiving a sensory stimulation program consisting of the stimulation of the five sensory modalities (tactile, gustatory, olfactory, auditory, visual senses) in a sequential order during 30-min session had a significantly better outcome than those in a control group (based on values of the Glasgow Coma Scale) [12]. In general, there are two basic therapeutic stimulation programs for DOC patients: the sensory stimulation approach and the sensory regulation approach [9]. The sensory stimulation approach is based on the assumption that intensive stimulation of all the senses will enhance synaptic re-innervation and stimulates the reticular activating system [7]. In contrast, the sensory regulation approach assumes that the capacity for information processing in these patients is limited. It therefore emphasizes the enhancement of selective attention by regulating the environment rather than providing high degrees of stimulation [13].

Mobilization into a standing position, e.g., with a tilt table, has been shown to improve arousal and awareness in small groups of VS and MCS patients [14–16]. Although desirable, it is not always easy to perform head-up tilt in the early phases of DOC. A common problem is the occurrence of syncopes due to a central sympathetic dysfunction and the absence of the venous pump due to paralyzed leg muscles [16]. To re-enforce cardiovascular response and to stabilize blood circulation, passive leg exercise to support the venous pump has been combined with head-up tilt by using a tilt table with an integrated robotic stepping device. Patients with DOC, analogous to patients with spinal cord injury, were shown to have reduced syncopes or presyncopes when using a tilt table with integrated stepping during mobilization [17,18]. This device made it possible to mobilize the patients into a standing position earlier and for a longer time during the course of rehabilitation. Assuming that the vertical position influences the state of arousal and alertness [14,15], an extension of verticality duration potentially enhances arousal and therewith the alertness and responsiveness of the patient.

This prompted us to design a prospective, single-blind, randomized controlled trial to determine the influence of earlier and increased mobilization by using a tilt table with integrated stepping. We hypothesized that 3 weeks of treatment using a tilt table with integrated stepping (compared to a conventional tilt table) could enhance recovery of consciousness in patients with DOC 4 weeks to 6 months post injury.

## Material and Methods

### Design

This study was a prospective, single-blind, stratified, randomized controlled trial with two intervention groups. According to the German Medical Devices Act (Medizinproduktegesetz), this study was registered at the German Institute of Medical Documentation and Information; DIMDI) prior to patient recruitment on June 27 2006 (reference number: AP4/3332/28/06). In addition and in order to satisfy international standards we also registered the study in Current Controlled Trials Ltd (www.controlled-trials.com) by August 31 2006, identifier number ISRCTN72853718. The authors confirm that all ongoing and related trials for this drug/intervention are registered. The study protocol was approved by the Ethics Committee of the Bavarian State Chamber of Physicians on May 30 2006, protocol number 06018. One modification to the initial protocol was made on December 22 2006 to change the age limit from 70 to 75 years. The study protocol and the informed consent form are available in English and German as supporting information files (S1 and S2 Protocols and S1 and S2 Files).

### Participants

All VS or MCS patients as defined by the Coma Recovery Scale-Revised (CRS-R) [4] who had been consecutively admitted to either the intensive care or the early rehabilitation unit of a neurological rehabilitation hospital were considered for inclusion in the study. The inclusion criteria were as follows: age 18–75 years, VS or MCS following traumatic brain injury, intracerebral bleeding or ischemic infarction, or MCS after hypoxic brain damage. Patients after hypoxic brain damage in VS were not included into the study. Patients were not enrolled in the study if any of the following criteria were met: (1) time since injury was less than 4 weeks or more than 6 months, (2) mobilization into standing lasted more than 30 minutes, (3) heart attack occurred within 4 weeks prior to admission, (4) patient wore a cardiac pacemaker, (5) severe condition of osteoporosis, (6) severe contracture or spasticity of the lower extremities were present; physical therapists rated the patient’s feasibility to participate in both study interventions based on their experience with tilt table and Erigo therapies, (7) unstable fractures or decubiti on lower extremities, (8) legal proxy refused to give written informed consent. All patients’ legal proxy signed a written informed consent form (study physicians enrolled patients).

### Sample Size

The number of patients was calculated a priori based on an analysis of a 6-week CRS-R time course of patients with early VS/MCS in our hospital. A mean improvement of 6.5 ± 3.5 points had been reported (unpublished data). As the time course and possible improvement of CRS-R values in a large group of VS/MCS patients had not yet been reported, an additional improvement by 3 points was considered clinically relevant. On the basis of 0.8 power for detecting a significant difference (p = 0.05, two-sided), 22 patients were required for each study group. In anticipation of possible dropouts, a total of 50 patients were enrolled.

### Outcomes

Primary outcome was the rate of improvement in the CRS-R score after the intervention. The CRS-R is a standardized neurobehavioral assessment tool comprising six hierarchically organized subscales measuring: auditory, visual, motor, oromotor–verbal function, communication, and arousal. The scores ranged from 0 to 23; higher scores indicated a higher level of neurobehavioral function [4]. Exhaustion from an intervention, bath, nutrition or another rehabilitation action might influence the alertness of a patient and consequently the results of the testing. In addition, the majority of patients with a severe DOC do not have a circadian sleep rhythm. Both aspects make it difficult to choose an optimal time for testing. To reduce this bias, the CRS-R was assessed twice at each measurement point; once on two separate days. The highest score was used for analysis.

Secondary outcome was determined by the modified Ashworth scale (MAS). The MAS grades the level of resistance encountered during manual passive stretching [19]. MAS values were collected for extensors and flexors of shoulders, elbows, wrists, hips, knees, and ankles on both sides, i.e., 24 values were obtained per patient. The MAS has moderate to good test-retest reliability but limited inter-rater reliability [20].

Both scores were assessed at baseline, after 3 weeks of treatment (week 3), and after 3 weeks of follow-up (week 6). Assessments of CRS-R and MAS were made by experienced therapists, one occupational therapist doing all CRS-R ratings, two physiotherapists all spasticity ratings. The assessors’ training consisted of repeated supervised practice of the assessments prior to the trial. The assessors were blinded with respect to the allocated intervention, but noted the presumed therapy on the assessment form.

### Study procedures

Treatment was assigned in random blocks by stratifying for type of brain injury (traumatic, non-traumatic) and state of consciousness by means of the CRS-R at the day of randomization (VS, MCS). These factors are shown to be predictive in terms of outcomes. Stratification was used to increase the comparability of both intervention groups [21,22]. The allocation of treatment groups was by a computer-generated random sequence provided by the University of Munich. Allocation sequence was thus concealed until interventions were assigned (the authors ML and CK assigned patients to the interventions). Participants were allocated to one of two groups: treatment on a conventional tilt table or on the Erigo.

The Erigo is a tilt table with an integrated robotic stepping device. The upper body of the patient is secured by a harness that fixes the chest and shoulder to the table. Leg movements are controlled by computer and are the result of passive thigh movement attained by securing the distal thigh to the device; the feet are strapped to two footplates. The inclination of the tilt table can be continuously adjusted from the horizontal to the vertical position. The speed of leg movement can be modified from 0 to 80 steps per minute [18].

After randomization the patients were scheduled for a total of ten sessions over 3 weeks, allowing 3 to 4 treatment sessions per week. The intervention was administered for 3 weeks, since it had been reported that at least 2 weeks of sensory stimulation were necessary for there to be any significant effect on consciousness [23]. Therapy sessions, both on the Erigo and the tilt table, were executed by two physical therapists or by one physical therapist with assistance provided for transfers from bed to device and back. Each session was scheduled for 1 hour gross therapy time. Net therapy time—starting after the patient was transferred to the device until the patient was returned to the supine position within the device–and the duration of the 70 degrees tilted head-up position were documented. Blood pressure, heart rate and oxygen saturation were monitored and an interruption of the verticalization procedure due to a syncope or presyncopal symptoms such as tachypnoea, tachycardia, pallor or increase in sweating was documented. The criteria for an interruption can be found elsewhere [18]. Patients were allowed to be further verticalized after an interruption occurred.

In addition to verticalization treatment, i.e., tilt table or Erigo, patients were treated by a dysphagia therapist, neuropsychologist, occupational therapist, and a physiotherapist. The therapists selected the content of the additional physiotherapy to meet the patient’s needs; however, prolonged mobilization out of bed into a standing position was not allowed, as this was the main focus of the study interventions. All patients received the same total amount of therapy (300 minutes therapy, incl. activating nursing, a day), as required by the German health care system.

### Data Analysis

For pre-test comparisons of demographic and outcome variables between the groups Student’s *t*-test, Mann-Whitney *U*-test, and *Chi* ^*2*^-analyses were used when appropriate. The respective tests used are reported in Table 1.

**Table 1:** Patient characteristics.

			Erigo group (n = 22)	Tilt table group (n = 22)	*p* value
**Age**			53 ± 15 (23–74)	52 ± 14 (23–71)	0.859 *
**Sex (f/m)**			10/12	8/14	0.760
**Time from injury to randomization [weeks]**			8 ± 3 (4–14)	8 ± 3 (4–14)	0.486 *
**Type of injury and CRS-R**	**non-traumatic**	**VS**	5	5	0.494
**category at randomization**	**non-traumatic**	**MCS**	10	14	
	**traumatic**	**VS**	3	1	
	**traumatic**	**MCS**	4	2	
**GCS at randomization**			9 (9–10)	9 (8–10)	0.605 ^#^

Values for ‘sex’ and ‘type of injury and CRS-R category at randomization’ are number of patients. ‘Age’ and ‘time from injury to randomization’ values are mean ± SD (range), ‘GCS’ values are *median* (25^th^– 75^th^ percentile). Abbr.: f, female; m, male; CRS-R, coma recovery scale-revised; VS, vegetative state; MCS, minimally conscious state; GCS, Glasgow coma scale. Statistical test was:

* *t*-test

^#^ *U*-test

without asterisk, chi^2^-test.

Patients with missing data on the CRS-R after the intervention (week 3) were not included in the analysis. The last value carried forward imputation method was used for data missing on the CRS-R follow-up measurement (week 6).

Non-parametric statistical methods are used for analysis to take into account the ordinal scale quality of the primary and secondary outcome parameters. To control for baseline values of the particular parameters, intra-individual differences (subtraction of value at baseline from value at week 3 and value at baseline from value at week 6) were calculated for between group comparisons (Erigo vs. tilt table) and analyzed using the Mann-Whitney *U*-test. Within group comparisons were analyzed using the Wilcoxon signed-rank test. Effect sizes for the non-parametric tests are reported as ‘r’ value. SPSS 17.0 was used for the statistical analyses. A difference was considered statistically significant if *p*<0.05.

## Results

**Fig 1:**
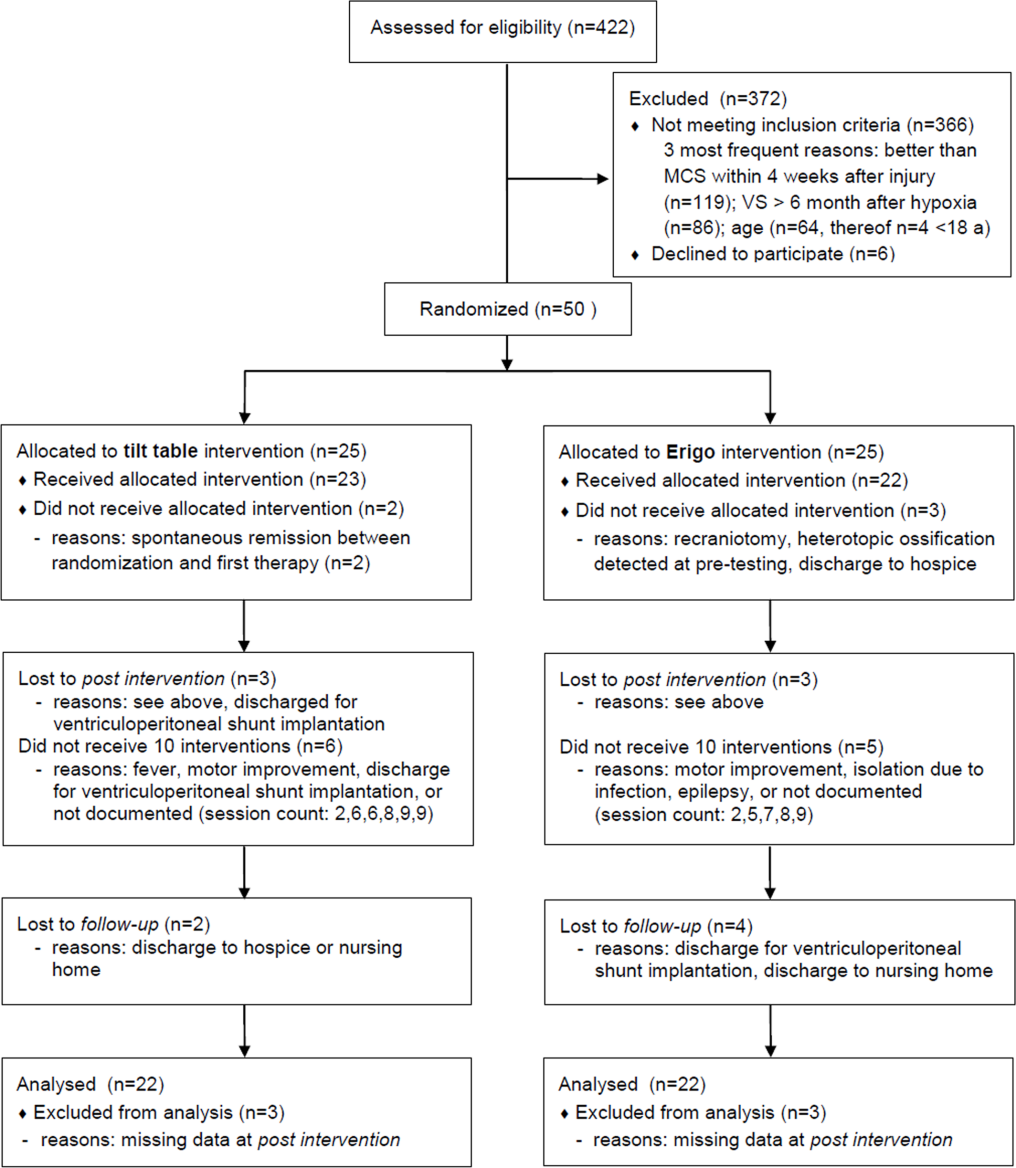
CONSORT flow chart.

Fifty patients were recruited for this study (first patient in: June 29 2006; last patient out September 02 2011). Fig 1 shows the flow of patients throughout the study (see CONSORT flow chart; a CONSORT checklist is available as S1 CONSORT Checklist).

The baseline characteristics of the treatment groups were similar, i.e., statistical analyses did not show significant differences. Values are summarized in Table 1. Patients in the Erigo group suffer from hypoxia (n = 3), subarachnoid hemorrhage (n = 11), or traumatic brain injury (n = 8). Patients in the tilt table group suffer from hypoxia (n = 6), subarachnoid hemorrhage (n = 9), traumatic brain injury (n = 3), or an ischemic stroke that causes a brain stem lesion (n = 4). A total of 12 patients (Erigo: 8; tilt table: 4) had a ventriculoperitoneal shunt. A valve reprogramming was only necessary in two cases, one in each group. During the study period 2 patients had a ventriculoperitoneal shunt implantation, which caused missing data (see CONSORT flow chart). Diabetes mellitus was present in 8 patients (Erigo: 5; tilt table: 3) and in 7 patients polyneuropathy was diagnosed (Erigo: 1; tilt table: 6). Individual patient characteristics can be found in S1 Table. Comparison of potentially confounding centrally-active medications during the six-week study period showed that dopaminergic agents and corticosteroids were used more often in the tilt table group, possibly increasing the outcome of the patients. On the other hand, amantadine hydrochloride, being the only agent showing evidence-based proved effectiveness in these patients, was used more often in the Erigo group. However, statistical analysis did not show any significant differences between both intervention groups (Table 2).

**Table 2:** Comparison of frequency of use of potentially confounding centrally-active medications during the six-week study period.

Drug class / name	Erigo	Tilt table	*p* value (chi^2^-test)
**Amantadine**	13	6	0.067
**Anidepressants (SSRI and Serotonergic/Noradrenergic)**	5	7	0.736
**Anticholinergics**	3	5	0.698
**Anticonvulsants**	7	8	1.000
**Antihistamines**	0	1	1.000
**Antihypertensive agents**	15	14	1.000
**Antispasticity agents**	6	6	1.000
**Atypical antipsychotics**	3	5	0.698
**Benzodiazepines**	3	2	1.000
**Beta blockers**	17	16	1.000
**Corticosteroids**	1	4	0.345
**Dopaminergic agents**	5	10	0.203
**Metoclopramide**	3	6	0.457
**Non-benzodiazepine hypnotics**	3	4	1.000
**Sympatomimetics**	1	3	0.607

### Primary outcome parameter CRS-R

**Fig 2:**
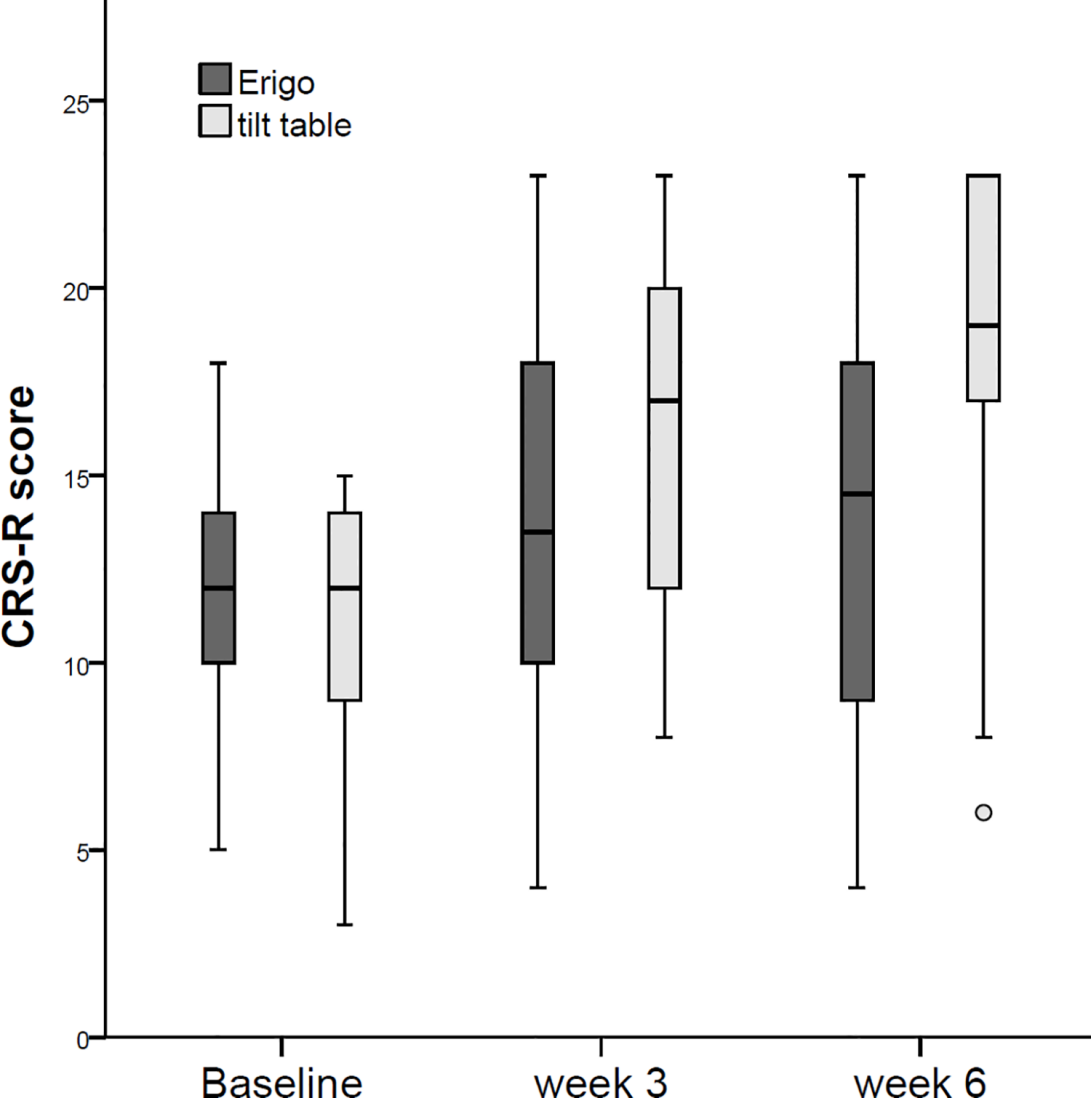
CRS-R values for the intervention groups.

Medians, 25^th^ and 75^th^ percentiles, and range for both intervention groups and the three time points of measurement are presented in Table 3 and Fig 2. The ‘last value carried forward’ method to refill missing data was required only in 4 patients of the Erigo group and in 2 patients of the tilt table group (see CONSORT flow chart, Fig 1).

**Table 3:** Coma Recovery Scale—Revised (CRS-R) at each time of measurement for both intervention groups.

	Total (n = 44)	Erigo (n = 22)	Tilt table (n = 22)
**Baseline**	12 (9–14, 3–18)	12 (10–14, 5–18)	12 (9–14, 3–15)
**Week 3**	16 (11–19, 4–23)	14 (10–18, 4–23)	17 (12–20, 8–23)
**Week 6**	18 (11–21, 4–23)	15 (9–18, 4–23)*	19 (17–23, 6–23)**

Values are *median* (25^th^– 75^th^ percentile, range). Abbr.: VS, vegetative state; MCS, minimally conscious state. Missing data were refilled with the last value carried forward imputation method:

* 2 cases

** 4 cases.

Values at baseline did not significantly differ between both intervention groups (*U*-test; *U* = 227.0, *z* = -0.354, *p* = .724, *r* = -.07). Using the Wilcoxon signed-rank test to evaluate changes over time within each intervention group, the analyses revealed significantly higher CRS-R values in week 3 and week 6 compared to the baseline values, both in the Erigo group (baseline—week 3: *z* = -2.177, *p* = .029, *r* = -.46; baseline—week 6: *z* = -2.194, *p* = .028, *r* = -.47) and the tilt table group (baseline—week 3: *z* = -4.003, *p* < .001, *r* = -.85; baseline—week 6: *z* = -4.003, *p* < .001, *r* = -.85). Indicating that both groups improved significantly during the intervention phase and that the gain in CRS-R maintained during the follow-up period. However, when comparing the intra-individual changes from baseline to week 3 between both interventions groups, the tilt table group was statistically significantly superior (median (25%-75% percentile); Erigo: 3 (0–5); tilt table: 4 (3–8); *U*-test; *U* = 144.5, *z* = -2.299 *p* = .021, *r* = -.34). The intervention effect was even more pronounced when analysing the change scores from baseline to week 6 (median (25%-75% percentile); Erigo: 4 (-1-6); tilt table: 9 (5–10); *U*-test; *U* = 122.0, *z* = -2.824, *p* = .005, *r* = -.42).

**Fig 3:**
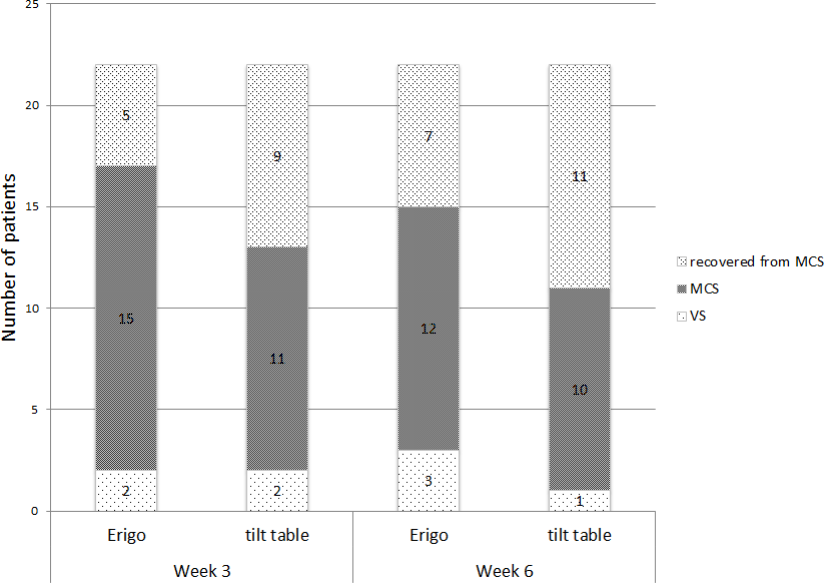
Number of patients in consciousness states.

At week 3, three patients in the Erigo group and 2 patients in the TT group were in VS, whereas 5 patients in the Erigo group and 9 patients in the TT group recovered and were no longer diagnosed as MCS. At week 6, three patients in the Erigo group and 1 patient in the TT group were in VS, whereas 7 patients in the Erigo group and 11 patients in the TT group were better than MCS. The maximum CRS-R score was reached in 4 patients (Erigo: 1; tilt table: 3) after the intervention phase, and in 8 patients (Erigo: 2; tilt table: 6) at the follow-up measurements. Data are also shown in Fig 3.

The analysis of the assessor’s assumption concerning the allocated intervention yielded following distribution for 38 ratings conducted: for the Erigo group the assessor assumed 11 patients to have received Erigo therapy and 7 patients to have received tilt table therapy. For the tilt table group the assessor assumed 13 patients to have received Erigo therapy and 7 patients to have received tilt table therapy. A chi^2^-test revealed following results: *χ* ^*2*^(1) = .062, *p* = .804. Thus, we can exclude an assessor bias, i.e., even in case the assessor had a preference for one of the two therapy methods, this bias would be equally distributed over both intervention groups.

Individual CRS-R scores can be found in S2 Table.

### Effects on spasticity

The modified Ashworth scale was used to detect spastic muscle tone. There was overall, a high frequency of missing data. Nearly half of all patients (47.7%) had missing data in at least one tested muscle group. The most common reason for this was muscle guarding. These patients were able to voluntarily move their extremities in a normal range of movement, indicating that no contracture or considerable increase in muscle tone could be present. However, when moved by the assessors, the passive movement was highly difficult to perform or showed rigidity. In this case no value was documented.

On the basis of the examined muscle groups, patients were categorized as having mild, moderate, or severe spasticity if MAS in at least one muscle group was ≥ 1and < 3, 3, or 4, respectively. At baseline 29 patients (Erigo, n = 14; tilt table, n = 15) had mild spasticity and 7 patients (Erigo, n = 4; tilt table, n = 3) had moderate spasticity, whereas none of the patients had severe spasticity in any of the tested muscles.

After the 3-week intervention period an improvement (reduction in spasticity) was found in 72 muscles, i.e., 8.5% of all MAS values and 45.6% of those values with an MAS score >0 at baseline. A worsening was found in 167 tested muscles (19.7% of all values) with a newly developed spasticity in 127 muscles, whereas 608 (71.8% of all values) did not show any difference. Group MAS changes, separated for upper and lower extremities can be found in Table 4. Comparison of intra-individual differences between the two intervention groups revealed no statistically significant difference (Mann-Whitney *U*-test: *U* = 86586; *Z* = -1.087; *p* = .277). Individual Ashworth scores can be found in S3 and S4 Tables.

**Table 4:** Changes in spasticity by means of the Modified Ashworth Scale.

	Erigo			Tilt table		
	worsening	no change	improvement	worsening	no change	improvement
upper extremity	12.2% (51)	34.3% (143)	5.0% (21)	13.0% (56)	29.3% (126)	5.6% (24)
lower extremity	5.5% (23)	39.6% (165)	3.4% (14)	8.6% (37)	40.5% (174)	3.0% (13)
Total	17.7% (74)	73.9% (308)	8.4% (35)	21.6% (93)	69.8% (300)	8.6% (37)

Values are % of the tested muscles per intervention group and (n).

Analysis of therapy documentation showed that net therapy times of the Erigo sessions was statistically significantly longer than those of the tilt table sessions (mean (SD) in minutes: Erigo, 25 (5), tilt table, 23 (8); *t*-test: *t* (388) = -2.671, *p* = .007, 95% CI: -3.177 - -.483) as well as the duration of the 70 degrees head-up tilt (mean (SD) in minutes: Erigo, 10.5 (6.5), tilt table, 7 (7); *t*-test: *t* (387) = -5.013, *p* < .001, 95% CI: -5.11 - -2.35). The number of interruptions in the tilt table group (91 interruptions) were significantly more frequent than in the Erigo group (26 interruptions) (chi^2^-test: *χ* ^*2*^(1) = 46.966, *p* < .001). In both groups the most frequent cause for an interruption was the occurrence of hypotension (Erigo: 57.7% of all Erigo interruptions; tilt table: 59.1% of all tilt table interruptions), followed by the occurrence of syncope or presyncopal symptoms (Erigo: 15.4% of all Erigo interruptions; tilt table: 32.3% of all tilt table interruptions). Individual therapy documentation can be found in S5 Table.

## Discussion

This randomized controlled clinical trial examined physical intervention methods used in the therapy of patients in minimally conscious or vegetative states following a traumatic or non-traumatic brain injury. These approaches involved use of either a conventional tilt table or a tilt table with an integrated robotic stepping device (Erigo). Both groups significantly improved during the 3-week intervention period. The Erigo group improved by a median of 2 points on the CRS-R. However, the rate of recovery of patients was better in the conventional tilt table group; these patients improved by a median of 5 points on the CRS-R and an additional 2 points during the 3-week follow-up period. On the basis of our sample size estimate this result might mean that patients receiving Erigo therapy were hindered in their rehabilitation process. On the other hand, our estimate might have been too optimistic, and the underlying patient sample might have been potentially biased by the routinely applied tilt table therapy. The results of the ‘German registry for coma outcome in patients undergoing acute rehabilitation‘ (KOPF-R) [24] revealed a mean improvement by 4 points on the CRS-R during a 3-month interval, indicating a 2-point improvement over 6 weeks. Thus, we assume that the Erigo therapy group showed typical improvement, whereas the conventional tilt table group had an additional benefit.

Nevertheless, our study apparently disproved the initial hypothesis that the recovery of consciousness in DOC patients could be enhanced by administering 3 weeks of treatment on a tilt table with integrated stepping compared to a conventional tilt table 4 weeks to 6 months post injury. The demographic data or the medications taken during the study period are not likely responsible for the difference in CRS-R outcome as there were no significant differences. Although a total of ten cases in the tilt table group could have a worse prognosis (6 patients with hypoxic etiology, 4 with brain stem lesions), the distribution for the factors shown to be predictive in terms of outcomes (traumatic/ non-traumatic and VS/ MCS) were sufficiently stratified [21,22]. In the present study the integrated stepping device improved the time that patients can be verticalized, as it has been shown in an earlier study [18]. Although the 2-minute difference in the net therapy time was statistically significant, the clinical significance is at least questionable bearing in mind the therapy durations of 25 and 23 minutes, respectively. The 4-minute difference for the duration of the 70 degrees tilt however represents an improvement of about 50% and thus seems not only statistically but also clinically significant. Thus–in contrast to our hypothesis—an extended verticalization achieved by a tilt table with an integrated stepping device did not lead to a better recovery in our patients.

In search for the reason of the better study outcome in the tilt table group we thoroughly examined further differences between our two treatment options. One striking difference is the abundance of sensory stimulation, which patients on the Erigo experience during treatment. Not only do cuffs move the patients’ legs, thus increasing proprioceptive sensory information, but also the auditory system was stimulated by the sound of the electrical drives of the Erigo system. Lehembre et al. and Sitt & King et al. confirmed previous findings of studies on resting-state EEG recordings in patients with DOC, which showed that EEG delta-band power increased with the severity of the disorder [25,26]. Recent functional imaging studies of the human brain also observed that the functional connectivity is reduced in regions of the default mode network (DMN) [27–29] or the weighted global connectivity is reduced [30]. Although the functions of the DMN remain a subject of debate, Mantini & Vanduffel concluded in their review of functional neuroimaging and electrophysiological studies that in addition to spontaneous cognition, a crucial function is the monitoring of the environment [31]. Although the DMN activity in MCS patients and especially in VS patients is reduced, it is the disintegration of cognitive processing rather than the absence of local brain activation that characterizes the loss of consciousness [30]. These two aspects, i.e., the increase in EEG delta-band power and the reduced activity of the DMN, indicate that sensory processing is slowed, and any further processing of incoming sensory information is limited to lower order brain structures. This might explain why severely affected DOC patients seem to benefit more from a therapy with reduced sensory activation, like the tilt table therapy. Further studies should verify this hypothesis by including neurophysiological measurements and functional imaging to better describe the investigated study population.

Patients with severe DOC are known to have high risk of increasing spasticity and there is evidence that frequent physical therapy may have a positive effect on the patient’s spasticity [32]. In our study both tilt table therapies did lead to an improvement or had no change in spasticity in most instances, whereas only 19.7% of the tested muscles worsened. Whether these changes in spasticity were influenced by the study interventions or the values display just the typical development over time in our study population, cannot be determined with our study design. However, the changes in spasticity showed no statistically significant differences between both intervention groups.

Our study has certain limitations. First, it was conducted at an inpatient rehabilitation center where the study interventions augmented the total rehabilitation program, e.g., routine nursing care and involvement of several therapeutic professions such as occupational, pharmacological, dysphagia, and physical therapies. However, the overall time of activation care as well as of occupational, physical, and dysphagia therapies was controlled and did not differ between the groups, although it was not possible to determine every single content and duration of the regular therapy program of each patient. Nevertheless we cannot rule out a bias by a difference in the associated therapies. Since the standard rehabilitation interventions continued during the study, we cannot determine the degree to which the benefits of the study interventions are independent of or synergistic with such standard treatments. In addition, with respect to our argumentation on sensory overflow the ratio of a study intervention session duration to the entire rehabilitation day should be considered. Second, the primary outcome parameter CRS-R is not validated for the use in non-TBI patients nor for the use over time with repeated measures. Third, as the study sample comprised patients in a non-chronic stage (< 6 months) of DOC, spontaneous recovery within the study period cannot be ruled out. Fourth, our findings can be generalized only to a limited extent, since patients in a VS of non-traumatic origin with poor prognosis were not included [21]. However, the prognostic criteria for such patients are currently undergoing a comprehensive revision. For example, recent studies on therapeutic hypothermia in patients following cardiopulmonary resuscitation reported improved neurological outcomes [33]. The thorough reevaluation of the previously established decision rules on the basis of studies of post-cardiac arrest patients not treated with hypothermia promises to have consequences for studies like ours [34].

## Supporting Information

S1 CONSORT Checklist CONSORT checklist. (PDF)

S1 File Informed Consent form–English version. (PDF)

S2 File Informed Consent form–German version. (PDF)

S1 Protocol Study protocol–English version. (PDF)

S2 Protocol Study protocol–German version. (PDF)

S1 Table Individual patient characteristics. Abbr.: TT, tilt table; f, female; m, male; TBI, traumatic brain injury; CRS-R, coma recovery scale-revised; VS, vegetative state; MCS, minimally conscious state; GCS, Glasgow coma scale.
(DOCX)

S2 Table Individual CRS-R scores. Abbr.: TT, tilt table. *, missing data refilled with the last observation carried forward method.
(DOCX)

S3 Table Individual Ashworth scores–upper extremities. Abbr.: MD, missing data.
(DOCX)

S4 Table Individual Ashworth scores–lower extremities. Abbr.: MD, missing data.
(DOCX)

S5 Table Therapy documentation. Abbr.: n.a., no therapy applied or not documented; interruption 0, no therapy interruption; interruption 1, therapy interruption necessary.
(DOCX)
