# Supplementary material for: Tilt Table Therapies for Patients with Severe Disorders of Consciousness: A Randomized, Controlled Trial
Source: PLoS One. 2015 Dec 1;10(12):e0143180. doi: 10.1371/journal.pone.0143180 (PMC4666666; doi:10.1371/journal.pone.0143180)
Supplement: S1 File — (PDF) [file pone.0143180.s002.pdf]

Patient Information and Statement of Consent to Participate in a Clinical Study

"The Effects of Erigo Therapy on Vigilance and the Cardiovascular System of Neurological Patients in the Vegetative or Minimally Conscious State "

Dear Patient, Dear Relatives,

Your family member/ dependent has a serious disorder of consciousness. The medical term for this condition is the vegetative or minimally conscious state, i.e., the dependent can only respond to external stimuli to a limited extent, in other words he/she can only occasionally express his/her will or communicate only with severe limitations.
In the course of the medical treatment and neurological examinations of your dependent, it became apparent that he/she is eligible to participate in the above-mentioned study. This informed consent form contains all relevant information about the purpose of the study and the potential individual benefits for your dependent. You are requested to read this study information carefully and to sign the informed consent form if you agree to all the points mentioned. If you have any further questions, we will be happy to answer them in a detailed conversation with you.
Participation in this study is voluntary. As the legal proxy you can withdraw your agreement to participation in the study at any time without having to gave your reasons and without affecting your dependent. Your decision will not affect your dependent's right to any current or future medical care to which the he/she is entitled.

Potential Benefits

The main goal of the neurological rehabilitation of patients with severe disorders of consciousness is to improve the conscious state, i.e., the ability to make contact and to improve reactions (more frequent, faster, and more direct) of those patients who already can in one way or another communicate with their surroundings.

For decades now the standard therapeutic measure for achieving this goal has been the mobilization of the patient into a standing position, for example, by using a tilt table or by passive movement of the patient in bed. However, the mobilization in the early phase for up to several months after a severe brain injury can be difficult. Many patients at first tolerate it only briefly, until the first signs of imminent fainting appear (cold sweat, etc.).This is caused by the failure of the circulatory system to react to the uprighting; it must be trained to react again.

In recent years an apparatus called the Erigo was developed. It consists of a tilt table with an integrated robotic stepping device, by which the legs of the patients are passively caused to make movements similar to normal walking. Depending on the patient's physical condition the inclination of the tilt table can be continuously adjusted from the horizontal to the vertical position, and the stepping mechanism can be used at several frequencies. The passive stepping movements in parallel with the uprighting of the patient promote the venous blood flow back to the heart, in other words the danger of fainting due to the uprighting is reduced. This

positive effect of the *Erigo* was observed in a study with healthy subjects as well as in patients in our hospital.
We expect the combination of uprighting the patient and the passive movement to have above all a synergistic effect on the patient's alertness, i.e., there is a greater improvement of the conscious state than occurs with the classical vertical board therapy without leg movements.

In addition we also expect the passive stepping movements to have prophylactic effects as regards thromboses, and contractures of the legs, spasticity, and potentially bone atrophy.

(Removed for publication)

Fig 1: Person on the *Erigo*.

Description of Study Procedures

- If you agree to have your dependent participate in this study, he/she will be randomly allocated to one of two treatment groups. The one group will receive a therapy with the Erigo while the second group will receive a classical tilt table therapy without passive movement of the legs. The allocation to one of two intervention groups is required to unequivocally ascertain the intervention effects by comparing the results of both intervention groups.
- We want to expressly point out that both intervention groups are expected to have positive effects on vigilance and circulatory stability of the patients.
- Dr. Luther will do an initial examination when the study starts. This examination includes measurement of skin response, ECG, and blood pressure, in case this was not already done during standard rehabilitation.
- The study interventions will be coordinated with all other therapies and will be entered in the daily therapy schedule. Generally the study interventions are scheduled for ca. 4x per week.
- Each therapy session will last approximately 30 minutes net therapy time. In total, including preparation and post processing, the gross therapy time is 1 hour.

- The intervention period will last 3 weeks, i.e., altogether the patient will receive 12 therapy sessions on the Erigo or on the tilt table. During this intervention period your dependent will additionally receive the standard care for patients in the vegetative or minimally conscious state at the Neurologische Klinik Bad Aibling. This consists of dysphagia therapy, neuropsychological therapy, occupational therapy, and physiotherapy.
- A follow-up examination will be scheduled for 3 weeks after the last study intervention session in order to test for the long-term effects of the different therapies.

Risks/ Discomforts

- In unfavorable conditions so-called presyncopes can occur during the therapy, i.e., the patient can have a sudden feeling of faintness, which very seldom leads to unconsciousness or fainting (syncope). In order to detect such feelings of faintness at the very first sign, qualified personnel will monitor the therapy, the ECG, blood pressure, and pulse during the whole period.. If a feeling of faintness occurs during the therapy, the Erigo is immediately turned off and the table returned to a horizontal position. As a rule the patient's condition improves in the next few minutes. The therapy is then terminated for this appointment and continued only on the following day.
- To avoid the danger of overstretching the muscles or tendons, the patient is carefully examined before the study begins and the magnitude of movement in the hip, knee, and foot joints is measured. The Erigo is then adjusted to the specific values so as not to exceed the measured magnitude.
- If, despite exercising due care, your dependent suffers a serious adverse event during the study interventions due to negligence of the hospital staff, the Neurologische Klinik Bad Aibling has a third party liability insurance to cover such events. The insurance sum for personal injuries amounts to 5,200,000 euros.

Measurements for Study Purposes Only

- ECG, oxygen saturation, and blood pressure during the study intervention sessions.
- Determination of muscle tone of legs and arms according to the Ashworth scale.

Possibility of Study being Ended Early

- The participation of your dependent in this study is voluntary. You may choose to stop participation in this study at any time for any reason. If you decide that your dependent should not be in this study, it will not affect his/her right to any current or future medical care to which your dependent is entitled.
- The treating physician will share any significant new findings on Erigo that become known during this study, which could be important for the dependent. This does not affect your right to withdraw from this study at any time for any reason.
- It can prove necessary in individual cases that the treating physician advises you to end participation in the study ahead of time or to interrupt participation. Potential reasons could be the following:
    a) The patient no longer fulfills the inclusion criteria of the study because his/her neurological situation has changed.
- Continuing therapy poses a danger to the patient due to the development of an additional disease, injury or pressure sores (decubitus).

- If there are signs that the patient is having circulation problems or other physical difficulties during the therapy, we will decide together the possibility of a premature ending of participation in the study.

Confidentiality

During treatment medical findings and personal information of the patient are collected and recorded. The recording of the data collected in the context of this clinical trial is first in the original documents or in the medical file, in which the physician had previously also made entries. Those data that are important for the clinical trial will in addition be pseudonymised and entered in a separate documentation sheet.
According to the German medical product law, the performance of a clinical trial is only allowed, if you are agreed with the recording of the patient data and their disclosure to the responsible health authorities (local control authorities and the federal health authorities, in special cases also foreign health authorities). The disclosure of data is without the transfer of any names. We may share your information with others, such as those who planned or work with us on this study, or to whom we are required to share information by law.
To ensure that all data are correctly transferred from the original documents/patient file into the documentation sheets, employees of the federal health authorities can compare the pseudonymised documentation sheets with the original documents. When you agreed to participation in this clinical trial you also released the trial physician from his/her nondisclosure obligation in the sense that an official of the health authorities who is obligated to nondisclosure can inspect the original documents made in the course of this clinical trial if it is necessary.

Questions

You should ask questions about anything you do not understand in this printed form. You have the right to ask questions about your participation in this study at any time. If you have any questions about the study or if you have any problems such as a study-related injury or concerns about your participation in this study, you may contact:

Dr. med. M. Luther, Tel.: 08061 / 903-521

Head of Physicians Dr. med. Dipl.-Psych. F. Müller, Tel.: 08061 / 903-0

Consent to Participate in This Study

Printed Name of Patient: ....

Date of birth: ....

I agree for the above-named patient to participate in the research study **"The Effectiveness of Erigo Therapy on Vigilance and the Cardiovascular System of Neurological Patients in the Vegetative or Minimally Conscious State “**.

I have been informed about aim, significance and course of the study as well as about possible burdens and risks by the written patient/test person information sheet that I received and by Mr./Mrs. ________________________. I have had time to ask questions and have been told whom to contact if I have additional questions now or in the future. I understand the possible risks and also that some risks may not be known.

By signing this form, I do not give up any legal rights.

You are responsible for providing truthful information about your medical history and current physical condition. The participation of your dependent in this study is voluntary. You may choose to stop participation in this study at any time for any reason. If you choose that your dependent is not in this study, it will not affect his/her right to any current or future medical care to which your dependent is entitled.

I declare that I am agreed that pseudonymised medical data recorded in the framework of this study in questionaires and on electronic media be made available to the responsible control authorities regional authorities of regional government, higher federal authorities (Federal Institute for Drugs and Medical Products, Bonn), the Ethics Commission and foreign authorities for monitoring the proper performance of the study as well as the evaluation of the study Moreover, I also declare that I am agreed that an authorized official of the responsible national and foreign regulatory authorities who is obligated to secrecy can have access to my personal data recorded by the study physician as far as this is necessary for evaluating the study. For these measures I release the study physician from his/her obligation to secrecy.
I furthermore declare that I am agreed that in legally predetermined cases (e.g., serious incidents) my name, address, and date of birth can be revealed to the responsible authorities.

I will be provided a copy of this consent form after I sign it. The original remains with the study physician.

....
(Date, Printed Name and Signature of Legal Proxy)

....
(Date, Printed Name and Signature of Investigator)
