# Supplementary material for: Tilt Table Therapies for Patients with Severe Disorders of Consciousness: A Randomized, Controlled Trial
Source: PLoS One. 2015 Dec 1;10(12):e0143180. doi: 10.1371/journal.pone.0143180 (PMC4666666; doi:10.1371/journal.pone.0143180)
Supplement: S2 File — (PDF) [file pone.0143180.s003.pdf]

# Patienteninformation und Einwilligungserklärung zur Teilnahme an der Studie

# "Die Auswirkungen der Therapie mit dem Erigo bei neurologischen Patienten mit apallischem Durchgangssyndrom oder im minimal responsiven Status auf Vigilanz und Kreislauf“

Sehr geehrte Patientin, sehr geehrter Patient, sehr geehrter Angehöriger,

Zur besseren Lesbarkeit wird im Folgenden auf die Doppelnennung von männlichen und weiblichen Formulierungen wie Patient/In, Arzt/Ärztin verzichtet und grundsätzlich nur eine Geschlechtsform verwendet.

Ihr Angehöriger befindet sich zur Zeit im Wachkoma, im medizinischen Sprachgebrauch als apallisches Syndrom bezeichnet, oder im sogenannten minimal responsiven Zustand, d.h. er kann nur eingeschränkt auf Reize von außen reagieren und seinen Willen nur manchmal oder nur eingeschränkt mitteilen. Zusammenfassend werden diese Krankheitsbilder auch als Bewusstseinsstörung bezeichnet.
Im Rahmen der klinischen Untersuchung hat sich herausgestellt, dass Ihr Angehöriger alle Voraussetzungen für die Teilnahme an der oben genannten Studie erfüllt. Diese Informationsblätter enthalten alle wesentlichen Angaben über die Ziele der Studie und über den möglichen persönlichen Nutzen für den Patienten. Wir möchten Sie bitten, diese Patienteninformation genau durchzulesen und, wenn Sie mit allen Punkten einverstanden sind, zu unterschreiben. Sollten Sie darüber hinaus Fragen haben, so sind wir gerne bereit, diese in einem ausführlichen Gespräch zu beantworten.
Wir möchten Sie ausdrücklich darauf hinweisen, dass die Teilnahme an einer klinischen Studie grundsätzlich freiwillig ist und jederzeit ohne Angabe von Gründen durch Sie beendet werden kann, ohne dass dem Patienten hierdurch Nachteile in der medizinischen Betreuung entstehen.

## *Was versprechen wir uns von der Studie ?*

Vorrangiges Ziel bei Patienten mit einer Bewusstseinsstörung ist die Verbesserung der Bewusstseinslage, d.h. zum Beispiel die Ermöglichung einer Kontaktaufnahme bei Patienten im Wachkoma bis hin zur Verbesserung der Reaktionen (häufigere, schnellere und gezieltere Reaktionen) bei Patienten, die bereits auf die eine oder andere Art Kontakt mit ihrer Umgebung herstellen können.

Hierfür wird im klinischen Alltag der neurologischen Rehabilitation seit Jahrzehnten die Mobilisation aus dem Bett mit Hilfe eines sogenannten Stehbretts und die passive Bewegung des Patienten im Bett eingesetzt. Dabei kann aber die Mobilisation aus dem Bett in der Frühphase bis einige Monate nach einer schweren Hirnschädigung schwierig sein. Viele Patienten vertragen die Mobilisation aus dem Bett am Anfang nur sehr kurz bis sich die Vorzeichen einer Ohnmacht einstellen (kalter Schweiß etc). Dies liegt an einer fehlenden Reaktion des Herzkreislaufsystems auf die Aufrichtung, die erst wieder trainiert werden muss.

In den letzten Jahren wurde ein Gerät, der sogenannte *Erigo*, entwickelt, bei dem ein Stehbrett mit einem Antrieb gekoppelt wurde, so dass die Beine des Patienten in einer dem natürlichen Laufen ähnlichen Art und Weise bewegt werden können. Das Stehbrett kann angepasst an die

aktuelle Belastungsfähigkeit unterschiedlich stark aufgerichtet werden und die Bewegungsgeschwindigkeit der Beine kann unabhängig vom Grad der Aufrichtung eingestellt werden. Durch die passive Bewegung der Beine beim Aufrichten wird dabei der venöse Blutrückstrom zum Herzen gefördert, d.h. die Gefahr einer Ohnmacht als Folge des Aufrichtens wird verringert. Dieser positive Effekt des *Erigo* wurde bei gesunden Probanden bereits in einer Studie belegt und in unserem Haus an Patienten beobachtet.

Durch die Kombination von Aufrichtung des Patienten und passiver Bewegung erwarten wir uns aber vor allem einen synergistischen Therapieeffekt auf die Wachheit des Patienten, d.h. eine schnellere Verbesserung des Bewusstseinszustandes als mit der klassischen Stehbretttherapie ohne Beinbewegungen.

Durch die Bewegung der Beine erwarten wir zusätzlich auch prophylaktische (vorbeugende) Effekte hinsichtlich Thrombosen, Gelenkeinsteifungen an den Beinen, Spastik und eventuell auch auf den Knochenabbau.

(Das Bild wurde für die Veröffentlichung entfernt.)

Abb 1: Proband auf dem *Erigo*.

### *Wie gestaltet sich der Therapieablauf ?*

- Falls Sie sich mit Unterzeichnung der Einverständniserklärung für eine Studienteilnahme entschieden haben, wird der Patient über ein Losverfahren einer von zwei Therapiegruppen zugeordnet. Die eine Gruppe wird eine Therapie mit dem *Erigo* erhalten, die andere Gruppe erhält eine klassische Stehbretttherapie, bei der der Patient ohne Bewegung der Beine aufgerichtet wird. Die Aufteilung in zwei Patientengruppen ist notwendig, um zweifelsfrei die Therapieeffekte des *Erigo* im Vergleich zur Standardtherapie feststellen zu können.

  Wir möchten aber ausdrücklich darauf hinweisen, dass wir uns bei beiden Therapieformen einen positiven Effekt auf Vigilanz (=Bewusstseinsgrad) und Kreislaufstabilität erwarten und somit beide Gruppen von der Behandlung profitieren.

- Vor Beginn der Studie wird Frau Dr. Luther den Patienten genau untersuchen. Dabei wird eine Messung der Hautleitfähigkeit, sowie EKG- und Blutdruckmessung durchgeführt, falls dies nicht ohnehin bereits durchgeführt wurde.
- Die Therapie erfolgt zu jeweils rechtzeitig abgesprochenen Terminen, die auf dem Therapieplan eingetragen sind, und findet in der Regel 4x wöchentlich statt.
- Eine einzelne Therapieeinheit dauert ca. 30 Minuten, incl. Vor- und Nachbereitung sollten Sie mit einer Gesamtzeit von etwa 1 Stunde rechnen.
- Die Studiendauer beträgt 3 Wochen, d.h. der Patient wird 12 Therapieeinheiten auf dem Erigo oder dem Stehbrett erhalten. Während dieser Zeit wird neben der Therapie im Rahmen der Studie natürlich das in der Neurologischen Klinik Bad Aibling für apallische und minimal responsive Patienten vorgesehene Therapieangebot aus aktivierender Pflege, Physio- / Ergo- und Schlucktherapie sowie Physikalischer Therapie durchgeführt.
- 3 Wochen nach Studienende ist eine Nachuntersuchung vorgesehen, in der Langzeiteffekte der unterschiedlichen Therapien auf den Bewusstseinszustand überprüft werden sollen.

### *Welche Risiken sind mit der Therapie verbunden?*

- Bei der Therapie kann es unter ungünstigen Bedingungen zu einer sogenannten Präsynkope kommen, d.h. einem Schwächeanfall, der in sehr seltenen Fällen bis zur Bewusstlosigkeit bzw. Ohnmacht (Synkope) führen kann. Um bereits die ersten Anzeichen eines solchen Schwächeanfalls zu erfassen, wird während der gesamten Zeit qualifiziertes Personal die Therapie überwachen und EKG, Blutdruck und Puls kontrollieren. Sollte ein Schwächeanfall während der Therapie auftreten, so wird der Erigo sofort abgeschaltet und die Liegefläche in die horizontale Position gebracht, worauf sich in der Regel der Zustand in den nächsten Minuten wieder bessert. Die Therapie wird dann für diesen Termin abgebrochen und erst am darauffolgenden Tag wieder fortgesetzt.
- Um Gefährdungen durch Überdehnung von Muskeln oder Sehnen zu vermeiden, wird der Patient vor Beginn der Studie sorgfältig untersucht und das Bewegungsausmaß an Hüft-, Knie- und Fußgelenk gemessen. Der *Erigo* wird dann so eingestellt, dass dieses ermittelte Bewegungsausmaß während der gesamten Therapie nicht überschritten wird.
- Sollte es trotz aller Sorgfalt durch die Therapie auf Erigo oder Stehbrett durch Verhalten unserer Mitarbeiter schuldhaft zu Schädigungen bei Ihrem Angehörigen kommen, wird dies durch die Haushaftpflichtversicherung der Neurologischen Klinik Bad Aibling abgedeckt, so dass die Kosten hierfür durch diese Versicherung gedeckt sind. Die Versicherungssumme für Personenschäden beläuft sich auf 5.200.000 Euro.

### *Welche Maßnahmen werden ausschließlich aus Studiengründen durchgeführt ?*

- EKG, Sauerstoffsättigungs- und Blutdruckmessung während der Therapie.
- Messung der Hautleitfähigkeit während der Therapie.
- Messung der Muskeltonuserhöhung der Beine und Arme mit Hilfe der Ashworth-Skala.

### *Wann wird Ihre Studienteilnahme vorzeitig beendet ?*

- Sie können jederzeit auch ohne Angabe von Gründen die Teilnahmebereitschaft widerrufen und aus der Studie ausscheiden, ohne daß dem Patienten dadurch irgendwelche Nachteile für die weitere medizinische Betreuung entstehen.
- Ihr behandelnder Arzt wird Sie über alle neuen Erkenntnisse umgehend informieren, die in Bezug auf diese Studie bekannt werden und für den Patienten wesentlich sein könnten. Sie

können daraufhin Ihre Entscheidung zur weiteren Teilnahme an dieser Studie neu überdenken.

- In Einzelfällen kann es notwendig sein, dass Ihr behandelnder Arzt Ihnen rät, die Teilnahme des Patienten an der Studie vorzeitig zu beenden oder zu unterbrechen. Die Gründe hierfür können sein:
  a) Der Patient erfüllt nicht mehr die Kriterien für die Studienteilnahme, weil sich die neurologische Situation verändert hat.
  b) Durch eine zusätzliche Erkrankung, Verletzung oder Druckgeschwür (Decubitus) liegt bei einer weiteren Therapie eine Gefährdung für den Patienten vor.
- Wenn es Hinweise gibt, dass der Patient während der Therapie Kreislaufprobleme oder sonstige körperliche Probleme hat, werden wir gemeinsam über einen vorzeitigen Studienabbruch entscheiden.

### *In welcher Weise werden die im Rahmen dieser klinischen Studie gesammelten Daten verwendet ?*

Während der Behandlung werden medizinische Befunde und persönliche Informationen des Patienten erhoben und niedergeschrieben. Die Aufzeichnung der im Rahmen dieser klinischen Prüfung erhobenen Daten erfolgt zunächst in Originalunterlagen bzw. in der Krankenakte, in der der Arzt auch bisher alle Befunde eingetragen hat. Die für die klinische Prüfung wichtigen Daten werden zusätzlich pseudonymisiert in einem gesonderten Dokumentationsbogen eingetragen.
Nach dem deutschen Medizinproduktegesetz ist die Durchführung einer klinischen Prüfung jedoch nur zulässig, wenn Sie mit der Aufzeichnung der Krankheitsdaten und deren Weitergabe an die zuständigen Gesundheitsbehörden (örtliche Überwachungsbehörden und Bundesgesundheitsbehörden, in Sonderfällen auch ausländische Gesundheitsbehörden) einverstanden sind. Diese Weitergabe erfolgt ohne Namensnennung.
Um sicherzustellen, daß alle Daten korrekt aus den Originalunterlagen/Krankenakte in die Dokumentationsbögen übertragen wurden, können die Eintragungen der pseudonymisierten Dokumentationsbögen durch Angehörige staatlicher Gesundheitsbehörden mit den Originalunterlagen verglichen werden. Mit Ihrem Einverständnis zur Teilnahme an dieser klinischen Prüfung entbinden Sie den Prüfarzt gegenüber den staatlichen Gesundheitsbehörden von seiner Schweigepflicht insofern, als ein zur Verschwiegenheit verpflichteter Beauftragter der Gesundheitsbehörden Einsicht in die im Rahmen dieser klinischen Prüfung erfolgten Originalaufzeichnungen nehmen kann, soweit zur Überprüfung der korrekten Datenübertragung notwendig.

### *Möglichkeit zur Besprechung auftretender Fragen*

Wir haben uns bei der Planung der Studie besonders bemüht, alle möglichen Eventualitäten in Betracht zu ziehen. Dies schließt nicht aus, dass es während der Therapie zu überraschenden Ereignissen kommt. In einem solchem Fall oder für weitere Fragen im Zusammenhang mit dieser Studie stehen Ihnen der behandelnde Arzt des Patienten und die Mitarbeiter an dieser Studie gerne zur Verfügung. Auch Fragen, die die Rechte des Patienten als Teilnehmer an dieser Studie betreffen, werden Ihnen gerne beantwortet.

Name der Kontaktperson: Dr. med. M. Luther, Tel.: 08061 / 903-521

Chefarzt Dr. med. Dipl.-Psych. F. Müller, Tel.: 08061 / 903-0

Einwilligungserklärung zur Teilnahme an der Studie

Name des Patienten in Druckbuchstaben: .................................................................

Geb. Datum: ............................

Ich stimme der Teilnahme von o.g. Patienten an der Studie **"Die Auswirkungen der Therapie mit dem Erigo bei neurologischen Patienten mit apallischem Durchgangssyndrom oder im minimal responsiven Status auf Vigilanz und Kreislauf“** freiwillig zu.

Ich bin über Sinn, Bedeutung und Verlauf der Studie sowie über mögliche Belastungen und Risiken anhand der schriftlichen Patienten-/Probanden-Information, die ich erhalten habe, und durch Herrn / Frau ________________________ aufgeklärt worden. In diesem Zusammenhang sind mir alle meine Fragen vollständig beantwortet worden.

Ich werde den ärztlichen Anordnungen, die für die Durchführung der Klinischen Studie erforderlich sind, Folge leisten, behalte mir jedoch das Recht vor, die freiwillige Mitwirkung jederzeit beenden zu können, ohne dass dem Patienten daraus Nachteile für seine weitere medizinische Betreuung entstehen.

**Ich erkläre mich damit einverstanden, dass im Rahmen dieser Studie erhobene Daten/Krankheitsdaten auf Fragebögen und elektronischen Datenträgern aufgezeichnet und ohne Namensnennung an die zuständigen Überwachungsbehörden (Landesamt oder Bezirksregierung), Bundesoberbehörde (Bundesinstitut für Arzneimittel und Medizinprodukte, Bonn), Ethik-Kommission und ausländischen Behörden zur Überprüfung der ordnungsgemäßen Durchführung der Studie sowie zur Bewertung von Studienergebnissen weitergegeben werden.**
**Außerdem erkläre ich mich damit einverstanden, dass ein autorisierter und zur Verschwiegenheit verpflichteter Beauftragter der zuständigen inländischen und ausländischen Überwachungs- und Zulassungsbehörden in meine beim Prüfarzt vorhandenen personenbezogenen Daten Einsicht nimmt, soweit dies für die Überprüfung der Studie notwendig ist. Für diese Maßnahme entbinde ich den Prüfarzt von seiner Schweigepflicht.**
**Ferner erkläre ich mich damit einverstanden, dass in gesetzlich festgelegten Fällen (z.B. schwerwiegende Zwischenfälle) mein Name, Anschrift und Geburtsdatum an die zuständigen Behörden weitergeleitet werden.**

Eine Kopie dieser Patienteninformation und Einwilligungserklärung habe ich erhalten. Das Original verbleibt beim behandelnden Arzt.

...........................................................................................................
(Datum, Name und Unterschrift des Betreuers / Bevollmächtigten)

...........................................................................................................
(Datum, Name und Unterschrift des verantwortlichen Arztes)
