# Supplementary material for: Tilt Table Therapies for Patients with Severe Disorders of Consciousness: A Randomized, Controlled Trial
Source: PLoS One. 2015 Dec 1;10(12):e0143180. doi: 10.1371/journal.pone.0143180 (PMC4666666; doi:10.1371/journal.pone.0143180)
Supplement: S1 Protocol — (PDF) [file pone.0143180.s004.pdf]

"The Effectiveness of Erigo Therapy on Vigilance and the Cardiovascular System of Neurological Patients in the Vegetative or Minimally Conscious State "

Investigators/ Trial Physicians:
Head of Physicians Dr. med. Dipl.-Psych. F. Müller
Dr. med. M. Luther

Principal Investigator:
Prof. Dr. med. E. Koenig

Neurologische Klinik Bad Aibling
Hospital Director: Prof. Dr. med. E. Koenig
Kolbermoorerstr. 72
83043 Bad Aibling
08061-903-0

Date/Version of study protocol
April 2006

1 Study overview

A major goal in the neurological rehabilitation of patients with severe disorders of consciousness is to enhance the arousal and ability of these patients to communicate.

To activate them and thus enhance their state of consciousness, patients are treated with multimodal stimulation. This stimulation consists of vestibular, auditory, tactile, olfactory, and visual inputs. To stimulate vestibular sensory information it appears to be essential to mobilize the patient into a vertical position. Although desirable, head-up tilt cannot always be easily realized in the early stages after brain injury when patients are still unconscious. A common problem is the occurrence of syncopes due to central sympathetic dysfunction and the absence of the venous pump as a result of paralysis of the leg muscles.

In the past, head-up tilt was performed gradually over a period of several weeks, in order to stabilize the patient’s circulatory system and prevent recurrent syncopes. In clinical practice this is done with a tilt table. The Erigo is a tilt table with an integrated robotic stepping device, which was developed to combine the advantages of a classical tilt-table therapy with those of a passive-movement therapy.. It provides a feasible way of simultaneously inducing passive leg movements and gradually inclined head-up tilt in patients who are comatose or semi-comatose. Moreover, it was shown that patients with a traumatic spinal cord injury with similar vegetative dysfunctions could be mobilized faster by using such a device.

The aim of this study is to determine, if an Erigo therapy has an immediate stabilizing effect on the cardiovascular system and if it increases vigilance in the early stages of mobilization of patients with a severe brain injury. These effects will be compared with a standard tilt-table therapy.

Vegetative functions will be determined by measuring blood pressure and recording heart rate before, during, and after the intervention. Reactions on the Erigo will be compared with those of the standard tilt table. In addition, vigilance will be measured by means of the Coma Recovery Scale-revised.

This study consists of two separate experimental procedures. The first is a pilot study with a cross-over design, in which the patients will be verticalized once on the Erigo and once on the traditional tilt table. The second is a single-blind randomized controlled trial, in which the above-named parameters for circulation and vigilance will be examined over a 3-week period. A follow-up examination will be scheduled 3 weeks after the last therapy. We would like to test the hypothesis that patients show a better effect on vigilance after the Erigo therapy than the patients who receive a tilt-table therapy.

2 Table of contents

1 Study overview 2
2 Table of contents 3
3 Introduction 4
4 Research question/Outcome criteria 5
4.1 Pilot study 5
4.2 Randomized controlled trial 5
4.2.1 Primary outcome measure 5
4.2.2 Secondary outcome measure 5
5 Medical device 6
5.1 General description 6
5.2 Effects (therapeutic, diagnostic) 7
5.3 Adverse events, risks, and stress for study participants 7
6 Study design/ Type of study 7
6.1 Method of randomization 8
6.1.1 Pilot study 8
6.2 Statistical analysis 8
6.2.1 Pilot study 8
6.2.2 Randomized controlled trial 8
6.3 Inclusion criteria 9
6.4 Exclusion Criteria 9
7 Study procedure 9
7.1 Procedure 9
7.2 Pre-enrollment procedure 10
7.3 Invasive measures 10
7.4 Non-invasive measures of pilot study 10
7.5 Non-invasive measures of randomized controlled study 11
8 Concomittant drugs 11
9 Adverse events/ Serious adverse events 11
9.1 Adverse event (AE) 11
9.2 Serious adverse event (SAE) 11
9.3 Recording and reporting 12
9.3.1 Recording an AE 12
9.3.2 Reporting and recording serious adverse events 13
9.4 Procedure during and after the study 13
10 Termination criteria 13
11 Ethical and legal regulations 13
11.1 Ethical regulations 13
11.1.1 Informed consent form 13
11.2 Legal regulations: Abidance by the law 14
11.2.1 Obligatory registration of the study 14
11.2.2 Permission of the Ethics Committee 14
11.2.3 Patient insurance 14
11.2.4 Data safety/ Confidentiality 14
12 List of medical products 14
13 Signatures 15
14 References 15

3 Introduction

A major goal in the neurological rehabilitation of patients in the vegetative or minimally conscious state is to enhance arousal and ability of the patients to communicate.

For decades patients have been treated with multimodal stimuli (vestibular, auditory, tactile, olfactory, and visual sensory inputs) to activate and thereby increase their state of consciousness. So far, there is no evidence that this treatment regimen enhances the rehabilitation outcome of patients ([1]).

However, some authors have reported changes in several parameters due to a stimulation, e.g., a significant change in heart rate and breathing rate during a tactile and auditory stimulation ([2]).

In general, the ascending reticular activating system (ARAS) plays an important role in activating energy for the entire brain and thus in any change in state of consciousness or the regulation of alertness and arousal. According to clinical experience mobilization into a vertical body position seems to play a crucial role in the enhancement of the patient’s vigilance. Neurons in the reticular formation and thus in the ARAS receive an excitatory and inhibitory sensory input, especially of the vestibular system.

In the early phase after an acute brain injury, it is frequently difficult to mobilize a patient into a sitting position, much less into a standing position, because of circulation problems due to vegetative dysfunctions resulting from the prolonged bed rest. Since the fifties it has been a therapeutic measure to verticalize the patient by steps on the tilt table in order to stabilize the circulation. However, a successful adaptation of the blood pressure to changes of position, which allows the patients to sit longer in a wheelchair, is frequently only possible after a period of weeks. ([3]).

A disorder of circulatory regulation in acute paraplegics is similarly pronounced due to the suppression of the sympathetic system. These patients have so far been mobilized with the tilt table. Recent studies in paraplegics showed that both the passive movement of the leg muscles ([4]) as well as activation of the leg muscles by means of functional electrostimulation ([5]) increase the circulation parameters of stroke volume and heart rate. Therefore the Erigo was developed, in order to combine the advantages of the classical tilt-table training with those of a movement therapy that is simple to perform. This device makes possible a passive movement of the legs with a smoothly adaptable degree of verticality. It was shown that healthy test persons had a significantly lower rate of neurocardiogenic syncopes during mobilization on the Erigo compared to the classical tilt-table therapy ([6]). A similar circulation-stabilizing effect can also be expected in patients with severe brain injury due to the reduction of venous pooling.

An earlier and longer mobilization in the course of rehabilitation and the simultaneous passive movement of the patients mean on the whole a faster mobilization and activation that hopefully finally leads to the more rapid achievement of the goal of rehabilitation.

4 Research question/Outcome criteria

4.1 Pilot study

The aim of the pilot study is to determine if patients in the vegetative or minimally conscious state potentially have a cardiovascular benefit from mobilization with the Erigo compared to therapy with a standard tilt table. The patient's circulation is expected to better adapt to the first verticalization, i.e., a prolonged mobilization in an extremely vertical position should be possible.
In addition, the state of consciousness will be examined to see if one therapy session on the Erigo can already improve the state of consciousness of the patients.
For this head control and muscle tone will be measured, as well as the reaction to a command. Further head control and muscle tone as well as a reaction to speech will be documented. In addition the efficacy of sensory stimuli will be measured by the changes in skin resistance and the variability of heart rate before and after therapy. The Coma Recovery Scale (CRS) will be applied during and after therapy.
If the above-described examinations can show that positive circulatory stabilization effects result from therapy on the Erigo, the mechanism underlying this success will be addressed in the next step. 'So far the neurological literature has assumed that passive leg movements already increase venous backflow to the heart ([4], [6]). It is, however, questionable from the internist's viewpoint, whether purely passive movements of a standing patient can lead to sufficient muscular pump action to prevent syncope.
A possible explanation is that spinal gait pattern generators are activated by means of the gait-similar movements on the Erigo and thus a reflexive, active co-movement of the patients' results ([7]). Therefore, a sample of treated patients will be examined using surface-EMG on different muscle groups of the leg to clarify if there is muscle activity during the Erigo therapy.

4.2 Randomized controlled trial

The same parameters will be collected in the randomized controlled trial as in the pilot study. We expect that a longer application of Erigo will have the result that the improved vigilance will be maintained after therapy is finished. Thus, the Coma Recovery Scale-Revised ([12]), as well as the skin conduction measurements and the heart rate variability will be collected before the intervention phase, and 3 and 6 weeks later. However, this will not be done during the interventions to ensure the blinding of the raters.

4.2.1 Primary outcome measure

To evaluate a clinically relevant change in the level of consciousness due to the Erigo therapy compared to the tilt-table therapy the following parameter will be measured:

- Coma Recovery Scale-Revised assessed by experienced, blinded rater

4.2.2 Secondary outcome measure

To evaluate an immediate stabilizing effect of the Erigo therapy on circulation, the following cardiovascular parameters will be measured:

- Heart rate
- Blood pressure; the Hypothesis: difference between initial value and the value after the intervention phase is greater for the tilt-table therapy (drop in blood pressure during tilt-table therapy)
- Oxygen saturation; the Hypothesis: increased oxygen saturation during Erigo therapy
- As co-variables the duration of verticalization (max. of 30 min per therapy session), and the degree of the maximum tilt will be analyzed

To evaluate a clinically unapparent effect on vigilance the following parameters will be measured:

- Skin conductance as reaction to command (should increase during Erigo therapy)
- Heart rate variability (should increase during Erigo therapy)
- Head control/movement during therapy (better during Erigo therapy)

The change in muscle tone or spasticity will be measured before the intervention, after the intervention, and after a 3 week follow-up period using the

- Ashworth-Scala.

To better compare the results of this study with results of international studies, the following parameters will be measured:

- at study onset: GCS (Glasgow Coma Scale)
- at discharge: GOS (Glasgow Outcome Scale)

5 Medical device

5.1 General description

Together with employees from the Orthopedic University Clinic Heidelberg and the Paraplegic Center in Balgrist (Zürich, Switzerland), a working group developed a so-called rehabilitation-stepper in order to combine the advantages of the classical standing board training with a simple to perform movement therapy. The relevant drive and positioning devices for the legs were integrated into a commercially available standing board, which is able to progressively adjust the degree of uprightness between lying and upright positions to the circulatory status of the patients. Thus, it is possible to move the legs in a way that corresponds to physiological walking (joint angle, phase-correct loading and unloading of the sole of the foot, speed). According to the latest neurophysiological findings, this type of movement provides a maximum trigger for activating the spinal gait pattern generators in the spinal cord and thus the muscles of the lower extremities ([7]).

(Photo removed for publication)

Figure 1: Erigo

5.2 Effects (therapeutic, diagnostic)

Potential Effects:

- Stabilization of the circulation – by improving the venous blood backflow to the heart by passive movement of the legs and activation of the muscles as a result of the triggering of the spinal gait program
- Improvement of vigilance – by simultaneously applying two forms of activation out of the coma stimulation

Additional expected therapy effects on secondary consequences of a long immobilization due to a severe quantitative disturbance of consciousness:
Osteoporosis prophylaxis – by means of phase-correct loading and unloading of bony structures

- Thrombosis prophylaxis – by passive movement of the legs and uprighting of the body
- Pneumonia prophylaxis – by increasing the intrathoracic gas volume by lowering the diaphragm during the upright positioning
- Contracture prophylaxis – by passive movement of the joint with adjusted speed
- Spasticity-reducing effect – through passive, reciprocal movement and activation of the spinal gait program

5.3 Adverse events, risks, and stress for study participants

- Under unfavorable conditions syncopes can occur during therapy on the Erigo. Possible risks include the loss of consciousness, prolonged bradycardia or hypotonia. However, we must point out that these orthostatic syncopes which occur in extreme cases can also occur in the transitional phase between lying and upright positioning during wheelchair mobilization, which takes place anyway, and the alternative tilt-table therapy. Since the passive leg movement on the Erigo occurs at the same time as the increased backflow of venous blood to the heart, the risk of syncope is according to the latest study information less on the Erigo than on the tilt table [6]). The remaining risks correspond to those of a typical physiotherapeutic treatment and thus are identical to those in the study and the control group. They are not increased by participation in a study compared to the usual therapy.

6 Study design/ Type of study

The pilot study is a prospective, monocenter, randomized study with a cross-over design. Each of the 20 participants will receive one therapy session on the Erigo and one on the tilt table. The time between the therapy sessions will last at least 1, maximally 7 days. The order of the type of treatment will be randomized.

The main study will be prospective, monocenter, controlled, and randomized. The main aim - to measure the improvement in vigilance on the Coma Recovery Scale – will be carried out by a blinded examiner. An experienced blinded examiner from the Neurological Clinic Bad Aibling will interpret the results. She will assess the data after their entry into a database, pseudonymization and randomized assignment to an intervention group. Depending on their group, the patients will receive four therapy sessions per week on the Erigo or on the tilt table. The study is planned to last three weeks. Irrespectively the normal range of therapy for apallic and minimally responsive patients (e.g., activating care, physio-, Ergo and swallowing therapy as well as physical therapy) will be performed. All study patients will receive at least five additional sessions of individual therapy besides the Erigo or tilt-table therapy.

A follow-up examination is planned to take place 3 weeks afte the end of therapy. In the three weeks between therapy end and the follow-up examination the patient will be mobilized by means of the classical physiotherapy, not with the Erigo or the tilt table. .

.

6.1 Method of randomization

6.1.1 Pilot study

Allocation procedure: All patients will receive one therapy session on the Erigo and one therapy session on the tilt table. The sequence of both therapies will be applied in random order. Consequently, patients will be randomized in one of two groups (1 = tilt table - Erigo, 2 = Erigo – tilt table) by means of a computer-generated randomization list.

Concealment: The computer-generated randomization list will be prepared by the Department for Medical Data Evaluation of the Schön Kliniken. **Study procedure:** Each patient who meets the inclusion or exclusion criteria will be immediately and consecutively reported on to the statistician or his/her representative. He/she will inform those involved in the project about each consecutive test person's group within the fixed order (descending) by telephone or in person and will note the patient data (Surname, Name, Date of birth) on the randomization list. The first patient will receive the number 1, the second patient, the number two, and so on, and group allocation will be recordet. A log book will be kept on the assignments and will be available for inspection at the end of the study. In this way the secrecy of the as yet not applied randomization order is ensured. A drop-out will be substituted by the next test person in chronological order, but the assignment to therapy and control group will be kept the same as that of the drop-out.

Randomized controlled trial

Allocation procedure: The study participants will be assigned to one of two groups: Group 1 = treatment with the tilt table, Group 2 = treatment with the Erigo. The randomized stratified grouping is performed in accordance with two criteria, which are expected to have an effect on the outcome of the patients: the original diagnosis (traumatic vs. non-traumatic) and severity of the disorder of consciousness according to the CRS at study begin (apallic vs minimally responsive) ([9]).

Non-disclosure conditions and study procedure are the same as in the pilot study.

6.2 Statistical analysis

6.2.1 Pilot study

The relative risk of a syncope occurring on the Erigo or the tilt table as well as the odds ratio will be calculated according to the statistics recommended.

6.2.2 Randomized controlled trial

Fifty patients will be enrolled in this study.

Sample size calculation

The sample size was estimated according to the main criterium measured by CRS and based on a 6-week follow-up on the effect of the standard therapy in 12 minimally responsive/apallic patients. The differential value of the CRS before and after a 6-week standard therapy showed a mean value improvement of 6.5 points with a variance of 12.3 (SD=3.5). An additional improvement of the CRS by 3 points is expected as results of the study planned here. This means that on the average an additional improvement in half of the evaluated items of the CRS is expected.

The sample size was estimated in order to check by inference statistical means the hypotheses of an interval-scaled feature ([10]) if the prerequisite of a corresponding normal distribution is fulfilled. The values α = 5% two-sided, power = 80% result in a group size of 22 patients for each of the two groups with the expected increase of CRS by 3 points. A difference of at least 3 points between the

two groups can be established as significant in case of a variance of the differential values (before/after treatment) of the CRS of $3.5^2 = 12.3$ and in group size of n=22 with an error probability of 0.5 and a power of 0.8. In addition the number of therapy drop-outs was so determined that the required number of patients will still be achieved even after subtracting an expected drop-out rate of 10%. Thus, a total of 25 patients as group size is included in the randomization procedure.
A 2-way analysis of variance (ANOVA) will be performed to statistically analyse the primary outcome parameter CRS-R. If the assumptions of homogeneity of variances or normal distribution are disproven, nonparametric tests will be used.

In case patients withdraw from the study at the wish of the caretaker, the patient will be referred to in the log book as “intention-to-treat”.

6.3 Inclusion criteria

- Condition following traumatic intracranial injury, hypoxic brain injury, intracerebral bleeding or ischemic stroke
- Apallic syndrome / minimally responsive state according to the CRS revised

  In the main study the category of patients with hypoxic brain injury will be restricted to minimally responsive patients, since various studies have shown a poor prognosis for apallic patients after a hypoxic brain injury ([11]). Thus their inclusion in a therapy study does not appear to be expedient. The acute event is not older than 6 months. The patient has so far been mobilized for less than 30 minutes in a standing position. The patient is not younger than 28 and not more than 70 years old.

6.4 Exclusion Criteria

- Acute cardiopulmonary diseases, i.e. pneumonia, bronchial asthma, heart attack, occurred within 4 weeks prior to study admission, etc.
- Instable fractures or decubiti on trunk and lower extremities
- Severe osteoporosis of the lower extremities
- Severe contractures of hip and knee
- Severe spasticity
- Pacemaker
- Pregnancy
- Leg length (heel to trochanter major) > 100 cm or < 70 cm.

7 Study procedure

7.1 Procedure

Erigo	Tilt table
Attachment of ECG electrodes, blood pressure cuff, and pulsoxymeter for continuous measurement of pulse, ECG, blood pressure, and oxygen saturation; also for performance of first measurement.	
Patient is transferred from bed/wheelchair to the Erigo or to the tilt table, is attached to it, and secured with a belt over the stomach.	

The foot supports are adjusted to the individual length of the legs of the patients.	
Adjustment of range of leg movement to patient´s maximally possible range.	
Start of leg drive at a maximally tolerated speed (the goal is 40 steps/min or at least 20 steps /min) while patient remains in horizontal position.	
Upright placement to 30 degrees; conditions maintained for about 5 min.	
Subsequently the patient is uprighted to 50 degrees (if appropriate, with same drive parameters); if circulation is good, to 70 degrees.	
At least 15-min-long or maximally 30-min-long therapy during the achieved angle of verticality. The duration of mobilization in the indicated time period is as long as possible, if there is no depression of the circulation and in agreement with the predetermined criteria (see below).	
At the end of therapy the Erigo or tilt table is lowered into the initial horizontal position by about 15 degrees, with a break for the patient to acclimatize him/herself. After the final measurement the apparatus is disassembled and the patient is transferred to the bed or wheelchair.	

7.2 Pre-enrollment procedure

The CRS-R values will be obtained before enrollment. An ECG will be recorded if none is available. Additional invasive measures will not be taken.

7.3 Invasive measures

No invasive measures are scheduled within the scope of the study.

7.4 Non-invasive measures of pilot study

Measures	Time point	Period	Frequency
Screening exam administered to determine eligibility, incl. use of patient medical record	pre enrollment	1 week	1x
ECG	pre enrollment	1 week	1x
ECG monitoring	during each therapy session	1 week	2x
Heart rate	during each therapy session	1 week	2x
Blood pressure	during each therapy session	1 week	2x
Oxygen saturation	during each therapy session	1 week	2x
Surface EMG	during each therapy session	1 week	2x
Skin conductance	before and after each therapy session	1 week	3x
Heart rate variability	before and after each therapy session	1 week	3x
Ashworth Scale	before and after each therapy session	1 week	2x
Coma Recovery Scale-Revised	before, during, and after each therapy session	1 week	6x
Glasgow Coma Scale	at enrollment	1 week	1x

7.5 Non-invasive measures of randomized controlled study

Measures	Time point	Period	Frequency
Screening exam administered to determine eligibility, incl. use of patient medical record	pre enrollment	6 weeks	1x
ECG	pre enrollment	6 weeks	1x
ECG monitoring	during each therapy	6 weeks	9x
Heart rate	during each therapy	6 weeks	9x
Blood pressure	during each therapy	6 weeks	9x
Oxygen saturation	during each therapy	6 weeks	9x
Skin response	before therapy, 3 and 6 weeks after start of therapy	6 weeks	3x
Heart rate variability	before therapy, 3 and 6 weeks after start of therapy	6 weeks	3x
Ashworth Scale	before therapy, 3 and 6 weeks after start of therapy	6 weeks	3x
Coma Recovery Scale-R	before therapy, 3 and 6 weeks after start of therapy	6 weeks	3x
Glasgow Coma Scale	before start of therapy	6 weeks	1x
Glasgow Outcome Scale	at hospital discharge		1x

8 Concomittant drugs

Drug therapies for internistic, urological or other comorbidities are fully permitted with the exception of those cited in the conditions of the exclusion criteria (acute pneumonia, bronchial asthma). As regards co-occurring diseases, decisions will be made on an individual basis, which will be noted in the study protocol and its interpretation commented upon.
A drug therapy with the goal of increasing vigilance should not be started during the study. If such a therapy already began before the patient entered our clinic or before he/she was enrolled in the study, then no change in medication should be made during the course of the study. Since sufficient proof for the success of a drug-induced increase in vigilance has not been presented by current clinical studies despite good theoretical models ([8]), it is ethically justifiable to not begin such therapy during the duration of the study.

9 Adverse events/ Serious adverse events

9.1 Adverse event (AE)

An AE is every unfavorable and unexpected event (including for example abnormal lab values), every symptom or every illness that occurs in a temporal context with the use of a drug, irregardless of whether a connection with the use of the drug is assumed or not. Especially every change in lab values or vital functions which occurs and requires discontinuation of the study application is to be considered an AE.

9.2 Serious adverse event (SAE)

Serious AEs (SAEs) are defined according to *The Trial Investigator`s GCP Handbook 1997 as* any untoward medical occurrences that:

- result in death,
- are life threatening (the participant must be at immediate risk of death from the event as it occurred; this definition does not include a reaction that, if it had occurred in a more serious form, might have caused death),

- require (or prolong) hospitalization,
- cause persistent or significant disability/incapacity,
- result in congenital anomalies or birth defects,
- lead to other conditions that in the judgement of the investigators represent significant hazards.

9.3 Recording and reporting

9.3.1 Recording an AE

Every AE must be documented in the report of adverse events.

In all cases the prinicipal investigator must be clearly identified in the AE report. The report must be dated and signed by him/her. The report should moreover contain an evaluation of the causal connection between the event and the drug. The report form must be completed
The description of the AE includes the time of its occurrence, the duration, severity, intensity, outcome and the relationship to the study application and the necessary treatment. The principal investigator should estimate the intensity of every AE on the basis of the following classification:

- **Mild:** the symptom is clinically visible but the participant does not require treatment or intervention, and the symptom does not affect the participant's clinical status.
- **Moderate:** the participant is symptomatic and requires treatment because the symptom affects his/her clinical status.
- **Severe:** the participant is symptomatic and requires intensive treatment because the symptom significantly diminishes his/her clinical status.

Therapeutic consequences must be documented, for example drug treatment, exclusion from the study.

An event is termed an "unexpected adverse event" in connection with the tested medical product (MP) if its nature or severity does not agree with the known data on the safety and tolerance of the MP.

Causality: The following definitions are used to determine the connection between the use of a medical product (MP) and the AE:

- **Safe:** A reaction that follows within a reasonable temporal course after use of the test product, that follows a known or expected response pattern to the suspected test product, and disappears when removed or when the dosage is reduced and recurs with repeated exposure.

- **Probable:** A reaction that follows within a reasonable temporal course after use of the test product, that follows a known or expected response pattern to the suspected test product, and disappears when removed or when the dosage is reduced and cannot be explained by the known features of the patient's clinical state.

- **Possible:** A reaction that follows within a reasonable temporal course after use of the test product, that follows a known or expected response pattern to the suspected test product, but which can be easily elicited by a number of other factors.

- **No connection:** A reaction for which there is sufficient information available to assume that there is no connection with the test product.

- **Unassessable:** It is not possible to assess a connection.

9.3.2 Reporting and recording serious adverse events

As soon as a severe AE is observed, even in the inclusion phase, the principal investigator writes an AE report. The report must clearly identify the principal investigator. The report must be dated and signed by the principal investigator. The report should also contain an evaluation of the causal connection between event and medical product.
Any interruption of the study participation is also to be immediately documented.

9.4 Procedure during and after the study

The principal investigator monitors the development of undesired clinical events or unhealthy lab values until the condition of the study participant improves or stabilizes.

10 Termination criteria

Individual

- If the systolic blood pressure falls by 20 mmHg or the diastolic blood pressure by more than 10 mmHg during the uprighting of the patient, therapy is continued at the next lowest tolerated angle of uprightness (30 or 50 degrees).
- The same procedure is followed with symptoms of a presyncope (tachypnoe, paleness, increased sweating…).
- Therapy is immediately terminated if the pulse falls below 40 beats/min or rises above 140 beats/min.
- Arterial hypotension (<80 mmHg).
- The occurrence of a new disease or complication of the primary illness that makes a continuation of the study impossible.
- Legal proxy withdraws participation agreement.

11 Ethical and legal regulations

11.1 Ethical regulations

The study will be conducted according to the Declaration of Helsinki and the ICH-GCP guidelines.

Participation in this study is voluntary. The legal proxy can withdraw his/her agreement to participate in the study at any time for any reason without affecting his/her dependent. If a legal proxy chooses that the patient not be in this study, it will not affect the right of the patient to any current or future medical care to which he/she is entitled.

11.1.1 Informed consent form

The study aim is to investigate tilt table/Erigo treatment of patients in the vegetative or minimally conscious state. These patients are not able to communicate in any structured conversation. Thus the study involves only patients who require legal care, except in case a medical care power of attorney is possessed
In the context of this study only therapeutic procedures or a combination thereof are used (verticalization and passive movement), which have been applied for centuries in early neurological rehabilitation. Although the data on these procedures from controlled clinical studies as regards the efficacy are insufficient, it is our opinion that withholding the stimulation methods from these patients – i.e., not applying them – is ethically questionable. Moreover, the risk of syncopes as undesired effects in the new therapy on the Erigo – presented above – is less than in the classic tilt-table therapy. All in all, a constancy or improvement of efficacy or reduction of risk is to be expected with the Erigo in comparison with the established tilt-table therapy.

Before enrollment the patient’s legal proxy will be informed verbally and in written form about the nature and character of the study, especially about its risks and benefits. Consent to participate will be given in written form by signing the informed consent form.

11.2 Legal regulations: Abidance by the law

This means that the regulations of the medical devices act (MDA) will be observed and the “Basic principles of the proper performance of the clinical trial of drugs“ as well as the Federal Data Protection Act (FDPA) will be applied.

11.2.1 Obligatory registration of the study

According to § 17 MPG (Medizinproduktegesetz, Medical Devices Act) this study will be registered with the relevant State Authority.

11.2.2 Permission of the Ethics Committee

The trial protocol will be approved by the Ethics Committee of the Bavarian State Chamber of Physicians before the start of the study. The study will not begin without the written permission of the Ethics Committee.

11.2.3 Patient insurance

The medical devices will be applied in accordance with their intended use. A separate insurance for the study patients is not necessary, as only approved medical devices will be used for all study procedures. In addition, all study procedures are non-invasive. In comparison with the standard rehabilitation interventions no additional risks are expected to result from study participation.
The Neurologische Klinik Bad Aibling has a third party liability insurance for residence purposes (insurance company: Bayerischer Versicherungsverband, Maximilianstr. 53, 80530 Munich, Versicherungsschein-Nr.: HV 77281 0500). This insurance covers all diagnostic and therapeutic interventions. The sum insured for personal injuries amounts to €5,200,000.

11.2.4 Data safety/ Confidentiality

The personal and other confidential information of patients are subject to the medical confidentiality and regulations of the Bundesdatenschutzgesetzes (Federal Data Protection Act). Any disclosure of patient data if necessary takes place only in anonymized form. If it is necessary in the course of the study to identify the name of a patient (monitoring), this is possible since the patients have expressed in writing their agreement that the principal investigator may be relieved of his nondisclosure obligation so that federal health officials can inspect the original patient files in order to check that the study is being properly conducted.

12 List of medical products

The Erigo used in the therapy is from the firm Hocoma; this is an approved medical device for use in the approved function (CE 0124). The tilt table and all medical devices for the above-planned examinations will be only medical devices that are used in their approved function, which is applied in the usual clinical operations of the Neurological Klinik Bad Aibling.

13 Signatures

Investigator/ Head Physician: Dr. med. Dipl.-Psych. F. Müller ______________________

Investigator/ Trial Physician: Dr. med. M. Luther ______________________

Principal Investigator: Prof. Dr. med. E. Koenig ______________________

14 References

[1] Lombardi F, Taricco M, De Tanti A, Telaro E, Liberati A, Sensory stimulation of brain-injured individuals in coma or vegetative state: results of a Cochrane systematic review. Clin Rehabil. 2002;16:464-72.

[2] Lippert-Gruner M, Wedekind C, Ernestus RI, Klug N: Early rehabilitative concepts in therapy of the comatose brain injured patients. Acta Neurochir Suppl. 2002;79:21-3.

[3] Welch R. Tilt-table therapy in rehabilitation of the trauma patient with brain damage and spinal injury. Nurs Clin North Am 1970; 5:621-30.

[4] Muraki S, Ehara Y, Yamasaki M: Cardiovascular responses at the onset of passive leg cycle exercise in paraplegics with spinal cord injury. Eur J Appl Physiol 2000; 81:271-74.

[5] Faghri PD, Yount JP, Pesce WJ, Seetharama S, Votto JJ., Circulatory hypokinesis and functional electric stimulation during standing in persons with spinal cord injury. Arch Phys Med Rehabil 2001; 82:1587-95.

[6] Czell D, Schreier R, Rupp R, Eberhard S, Colombo G, Dietz V: Influence of passive leg movements on blood circulation on the tilt table in healthy adults. Neuroengineering Rehabil. 2004 Oct 25;1(1):4.

[7] Duysens J., Van de Crommert H, Neural Control of locomotion: Part 1: The central pattern generator from cats to humans. Gait & Posture 1998; 7:131-141.

[8] Richer E, Tell L: Indications, efficacy and tolerance of drug therapy in view of improving recovery of consciousness following a traumatic brain injury. Ann Readapt Med Phys. 2003; 46:177-83.

[9] The Multi-Society Task Force on PVS: Medical Aspects of the Persistent Vegetative State (First of Two Parts). N Engl J Med 1994; 330:1499-508.

[10] Kalmar K, Giacino J. The JFK Coma Recovery Scale: An Ordinal or Interval Measure? Arch Phys Med Rehabil 2000; 81:1619.

[11] The Multi-Society Task Force on PVS: Medical Aspects of the Persistent Vegetative State (Second of Two Parts). N Engl J Med 1994; 330:1572-9.

[12]) Giacino J, Kalmar K, Whyte J. The JFK Coma Recovery Scale-Revised: Measurement Characteristics and Diagnostic Utility. Arch Phys Med Rehabil 2004; 85:2020-9.
