# Supplementary material for: Tilt Table Therapies for Patients with Severe Disorders of Consciousness: A Randomized, Controlled Trial
Source: PLoS One. 2015 Dec 1;10(12):e0143180. doi: 10.1371/journal.pone.0143180 (PMC4666666; doi:10.1371/journal.pone.0143180)
Supplement: S2 Protocol — (PDF) [file pone.0143180.s005.pdf]

"Die Auswirkungen der Therapie mit dem Erigo bei neurologischen Patienten mit apallischem Durchgangssyndrom oder im minimal responsiven Status auf Vigilanz und Kreislauf“

Studienverantwortliche Ärzte:
Chefarzt Dr. med. Dipl.-Psych. F. Müller
Dr. med. M. Luther

Projektleiter:
Prof. Dr. med. E. Koenig

Neurologische Klinik Bad Aibling
Ärztlicher Direktor Prof. Dr. med. E. Koenig
Kolbermoorerstr. 72
83043 Bad Aibling
08061-903-0

Datum/Version des Untersuchungsplans
April 2006

1 Zusammenfassung

Vorrangiges Ziel in der neurologischen Frührehabilitation von Patienten im apallischen Durchgangssyndrom oder im minimal responsiven Status nach schwerer Hirnschädigung ist das Wiedererlangen der Kontaktfähigkeit. Durch eine multimodale Stimulation der Patienten soll deren Aktivierung und damit eine Verbesserung des Bewusstseinszustandes erreicht werden. Die multimodale Stimulation beinhaltet dabei vestibuläre, akustische, kinästhetisch-taktile, olfaktorische und visuelle Reize. Ein entscheidender Faktor zur Vigilanzverbesserung scheint dabei als vestibulärer Reiz die Mobilisation in die Vertikale zu sein. Dabei ist gerade in der Frühphase nach dem Akutereignis die Mobilisation in den Sitz und v.a. in den Stand durch Kreislaufschwierigkeiten aufgrund von vegetativen Funktionsstörungen erschwert.

Als therapeutische Maßnahme zur möglichst zügigen Kreislaufstabilisierung dieser Patienten dient die stufenweise Vertikalisierung, um nach langer Bettlägerigkeit die autonomen Regulationsmechanismen wieder in Gang zu setzen.
Seit den 50-er Jahren wird im klinischen Alltag das Stehbretttraining zur Stabilisierung des Kreislaufs angewandt. Um die Vorteile des klassischen Stehbretttrainings mit einer einfach durchzuführenden Bewegungstherapie zu kombinieren, wurde der sogenannte Erigo entwickelt. Mit diesem Gerät ist die passive Bewegung der Beine bei stufenlos anpassbarem Grad der Aufrichtung möglich. Bei Patienten mit traumatischem Querschnitt, bei denen ähnliche vegetative Funktionsstörungen aufgrund einer Sympathikolyse vorliegen, gibt es bereits Erfahrungen, die auf eine schnellere Mobilisation durch Verwendung eines solchen Gerätes hinweisen.

Ziel dieser Studie ist nachzuweisen, ob für Patienten nach schwerer Hirnschädigung durch die Therapie mit dem Erigo eine direkte kreislaufstabilisierende Wirkung in der frühen Mobilisation sowie eine Verbesserung der Vigilanz erreicht werden kann. Dazu soll ein Vergleich mit der klassischen Stehbretttherapie durchgeführt werden.
Im Rahmen der Studie sollen mittels Messung von Blutdruck und Herzfrequenz die vegetativen Funktionen vor, während und nach einer Therapie auf dem Erigo untersucht und mit den Reaktionen auf dem Stehbrett verglichen werden. Zudem werden verschiedene Parameter zur Überprüfung der Vigilanz, wie z.B. die Coma Recovery Scale, festgehalten.
Die Studie ist in zwei Schritten geplant. Begonnen wird mit einer Pilotstudie im Cross-over Design, in der die Patienten jeweils einmal auf Erigo und Stehbrett mobilisiert werden. Im Anschluss werden die o.g. Parameter von Kreislauf und Vigilanz in einer kontrollierten, randomisierten und verblindeten Studie über einen Zeitraum von 3 Wochen erhoben. Eine Nachuntersuchung ist 3 Wochen nach Therapieende vorgesehen. Hierdurch soll die Hypothese überprüft werden, dass durch die Erigo-Therapie eine größere Vigilanzverbesserung in dieser Patientengruppe erreicht werden kann als mit der klassischen Stehbretttherapie.

2 Inhaltsverzeichnis

1 Zusammenfassung ... 2
2 Inhaltsverzeichnis ... 3
3 Einleitung / Wissenschaftliche Grundlagen ... 4
4 Ziele der Studie/Zielkriterien ... 5
4.1 Pilotstudie ... 5
4.2 Hauptstudie ... 5
4.2.1 Hauptzielkriterium ... 5
4.2.2 Nebenzielkriterien ... 5
5 Zu prüfendes Verfahren ... 6
5.1 Allgemeine Beschreibung ... 6
5.2 Wirkungen (therapeutisch, diagnostisch) ... 7
5.3 Unerwünschte Wirkungen, sonstige Risiken, Belastungen für den Studienteilnehmer ... 8
6 Studiendesign(-typ) ... 8
6.1 Randomisierungsverfahren/ggf. –plan ... 8
6.1.1 Pilotstudie ... 8
6.1.2 Hauptstudie ... 9
6.2 Statistisches Design ... 9
6.2.1 Pilotstudie ... 9
6.2.2 Hauptstudie ... 9
6.3 Einschlußkriterien ... 10
6.4 Ausschlußkriterien ... 10
7 Studienablauf ... 10
7.1 Versuchsdurchführung ... 10
7.2 Voruntersuchungen ... 11
7.3 Invasive Untersuchungen ... 11
7.4 Nichtinvasive Untersuchungen in der Pilotstudie ... 11
7.5 Nichtinvasive Untersuchungen in der Hauptstudie ... 12
8 Medikamentöse Begleittherapie ... 12
9 Definition unerwünschter Ereignisse ... 12
9.1 Definition eines Unerwünschten Ereignisses (UE/AE) ... 12
9.2 Definition eines Schwerwiegenden Unerwünschten Ereignisses (SUE/SAE) ... 12
9.3 Dokumentation und Meldung ... 13
9.3.1 Dokumentation eines unerwünschten Ereignisses: ... 13
9.3.2 Meldung und Dokumentation eines Schwerwiegenden Unerwünschten Ereignisses ... 14
9.4 Procedere während und nach der Studie ... 14
10 Abbruchkriterien ... 14
11 Ethische und rechtliche Aspekte ... 14
11.1 Ethische Grundlagen ... 14
11.1.1 Patienten-/Probandeninformation/Einverständniserklärung ... 14
11.2 Rechtliche Grundlagen: Einhaltung der Vorschriften des MPG, DSG ... 15
11.2.1 Meldung der Prüfung ... 15
11.2.2 Votum der Ethikkommission ... 15
11.2.3 Patienten-Versicherung ... 15
11.2.4 Datenschutz/Einblick in Originalkrankenunterlagen ... 15
12 Liste der verwendeten Medizinprodukte ... 16
13 Unterschriften ... 17
14 Literatur ... 17

3 Einleitung / Wissenschaftliche Grundlagen

Vorrangiges Ziel in der neurologischen Frührehabilitation von Patienten mit apallischem Durchgangssyndrom oder im minimal responsiven Status nach schwerer Hirnschädigung ist das Erlangen der Kontaktfähigkeit. Zur Aktivierung der Patienten wird dabei aufgrund der klinischen Erfahrung seit Jahrzehnten die multimodale Stimulation (vestibuläre, akustische, kinästhetisch-taktile, olfaktorische und visuelle Reize) eingesetzt, auch wenn bislang kein verlässlicher Beweis für ein hierdurch verbessertes Outcome erbracht werden konnte ([1]). Verschiedene Autoren beschreiben jedoch Veränderungen einzelner Parameter durch die Stimulation, so z.B. eine signifikante Veränderung in Herz- und Atemfrequenz bei taktiler und akustischer Stimulation ([2]). Für eine Veränderung der Bewusstseinslage ist u.a. das aktivierende aufsteigende reticuläre System (ARAS) in Form einer „Weckfunktion" kortikaler Strukturen verantwortlich Ein entscheidender Faktor zur Vigilanzverbesserung scheint deshalb - auch entsprechend der klinischen Erfahrung - die Mobilisation in die Vertikale zu sein, da die Formatio reticularis und damit das ARAS den größten Anteil hemmender und fördernder Impulse vom Vestibularissystem erhält.

In der Frühphase nach akuter Hirnschädigung und infolge einer längeren Bettlägerigkeit ist die Mobilisation in den Sitz und v.a. in den Stand jedoch häufig durch Kreislaufschwierigkeiten aufgrund von vegetativen Funktionsstörungen erschwert. Als therapeutische Maßnahme zur möglichst zügigen Kreislaufstabilisierung von Patienten dient seit den 50-er Jahren die stufenweise Vertikalisierung auf dem Stehbrett. Eine Adaptation des Blutdruckes bei Lagewechsel, die es den Patienten ermöglicht, auch längere Zeit im Rollstuhl zu sitzen, erfolgt jedoch trotz dieser Therapie häufig erst nach Wochen ([3]).

Die Kreislaufregulationsstörung ist dabei bei akuten Querschnittspatienten aufgrund der Sympathikolyse ähnlich ausgeprägt, auch bei ihnen wurde bislang die Mobilisation mit Hilfe des Stehbrettes durchgeführt. In neuester Zeit wurden Studien an querschnittgelähmten Patienten durchgeführt, die zeigen konnten, dass sowohl passives Durchbewegen der Beine ([4]) als auch eine Aktivierung der Beinmuskulatur mittels Funktioneller Elektrostimulation ([5]) zu einer Erhöhung der Kreislauffunktionsparameter Schlagvolumen und Herzfrequenz führt. Um deshalb die Vorteile des klassischen Stehbretttrainings mit einer einfach durchzuführenden Bewegungstherapie zu kombinieren, wurde der sogenannte Erigo entwickelt. Mit diesem Gerät ist eine passive Beinbewegung bei stufenlos anpassbarem Grad der Aufrichtung möglich. Bei gesunden Probanden konnte eine signifikant geringere Rate an neurokardiogenen Synkopen bei Mobilisation auf dem Erigo im Vergleich zur klassischen Stehbretttherapie gezeigt werden ([6]). Eine ähnlich kreislaufstabilisierende Wirkung durch eine Verminderung des venösen Pooling ist auch bei Patienten mit schwerer Hirnschädigung zu erwarten.

Durch eine im Rehabilitationsverlauf frühere und längere Mobilisation sowie die zeitgleiche passive Bewegung des Patienten ist somit insgesamt eine schnellere Mobilisation und Aktivierung sowie letztendlich ein schnelleres Erreichen des Rehabilitationszieles zu erhoffen.

4 Ziele der Studie/Zielkriterien

4.1 Pilotstudie

In der Pilotstudie soll vorrangig die Frage beantwortet werden, ob die Mobilisation mit dem Erigo eine größere kreislaufstabilisierende Wirkung bei apallischen / minimal responsiven Patienten im Vergleich zu einer klassischen Stehbretttherapie entwickelt, dass heißt, ob schon in der ersten Mobilisation eine bessere Adaptation des Kreislaufes, d.h. eine längere Mobilisation bei hohem Aufrichtwinkel erreicht werden kann.

Zusätzlich soll untersucht werden, ob bereits durch eine einmalige Therapie auf dem Erigo eine Verbesserung des Bewusstseinszustandes der Patienten erreicht werden kann. Hierzu dienen zum Einen die Dokumentation von Kopfkontrolle und Muskeltonus, sowie einer Reaktion auf Ansprache. Zum Anderen werden als Maß der Wirksamkeit sensorischer Reizangebote Hautwiderstandsveränderungen und Herzratenvariabilität vor und nach Therapie gemessen. Jeweils vor, während und nach Therapie erfolgt die Durchführung der Coma Recovery Scale (CRS).

Wenn sich in den oben beschriebenen Untersuchungen zeigen lässt, dass durch die Therapie auf dem Erigo eine Kreislaufstabilisierung erreicht werden kann, soll in einem nächsten Schritt versucht werden, den Mechanismus dieses Erfolges zu klären. Bislang wird in der neurologischen Literatur davon ausgegangen, dass passive Beinbewegungen bereits den venösen Rückstrom zum Herzen erhöhen ([4], [6]). Aus internistischer Sicht ist aber fraglich, ob eine rein passive Bewegung beim stehenden Patienten zu einer ausreichenden Muskelpumpe führen kann, um Synkopen zu vermeiden.
Eine mögliche Erklärung ist deshalb die Aktivierung des spinalen Gangmustergenerators durch die gangähnliche Bewegung auf dem Erigo und somit eine reflektorische aktive Mitbewegung des Patienten ([7]). In einer Untersuchung an einer Stichprobe der behandelten Patienten soll deshalb mit Hilfe eines Oberflächen-EMG an verschiedenen Muskelgruppen des Beines geklärt werden, ob es während der Erigo-Therapie zu einer Muskelaktivität kommt.

4.2 Hauptstudie

In der Hauptstudie werden die selben Parameter erhoben wie in der Pilotstudie. Allerdings erwarten wir bei längerer Anwendung des Erigo ein Anhalten der verbesserten Vigilanz über die Therapie hinaus, so dass sowohl Coma Recovery Scale-Revised ([12]) als auch Handwiderstandsmessung und Herzratenvariabilität zu Beginn der Therapie und zu den Zeitpunkten drei und sechs Wochen nach Therapiebeginn erhoben wird, nicht jedoch während der Therapien. Hierdurch wird zudem eine Verblindung des Untersuchers möglich.

4.2.1 Hauptzielkriterium

Zum Nachweis einer für den Patienten klinisch relevanten Verbesserung durch eine Therapie mit dem Erigo im Vergleich zur klassischen Stehbrett-Therapie wird die

- Coma Recovery Scale-Revised durch einen erfahrenen, verblindeten Beurteiler erhoben.

4.2.2 Nebenzielkriterien

Zum Nachweis der direkten kreislaufstabilisierenden Wirkung des Trainings mit dem Erigo werden folgende Kreislauffunktionsparameter erhoben:

- Herzfrequenz
- Blutdruck, Hypothese: die Differenz zwischen Wert zu Beginn und am Ende der Therapie ist im Stehbrett höher (Blutdruckabfall im Stehbrett)
- Sauerstoffsättigung, Hypothese: verbesserte Sauerstoffsättigung unter Erigo-Therapie

- Als Covariable geht die Dauer der möglichen Mobilisation (durch äußere Rahmenbedingungen max. 30 Minuten/Therapieeinheit) sowie der erreichte maximale Aufrichtwinkel in die Auswertung mit ein.

Als Parameter einer klinisch noch nicht apparenten vigilanzfördernden Wirkung werden folgende Variablen erfasst:

- Hautwiderstandsveränderung auf Ansprache (Zunahme bei Erigo-Therapie)
- Analyse der Herzfrequenzvarianz (Zunahme bei Erigo-Therapie)
- Kopfkontrolle unter Therapie (Zunahme bei Erigo-Therapie)

Der Verlauf des Muskeltonus, respektive die Entwicklung oder Verringerung einer bereits bestehenden spastischen Muskeltonuserhöhung wird zu Beginn, nach 3 Wochen und nach Abschluss der Studie mit der

- Ashworth-Skala erhoben.

Zur besseren Vergleichbarkeit mit internationalen Studiendaten wird

- bei Studieneintritt die GCS (Glasgow Coma Scale) und
- bei Entlassung aus der stationären Behandlung die GOS (Glasgow Outcome Scale) erhoben.

5 Zu prüfendes Verfahren

5.1 Allgemeine Beschreibung

Um die Vorteile des klassischen Stehbretttrainings mit einer einfach durchzuführenden Bewegungstherapie zu kombinieren, wurde von einer Arbeitsgruppe mit Mitarbeitern aus der orthopädischen Universitätsklinik Heidelberg sowie dem Paraplegikerzentrum in Balgrist (Zürich) ein sogenannter Reha-Stepper entwickelt: In ein kommerziell erhältliches Stehbrett, bei dem der Grad der Aufrichtung stufenlos zwischen liegender und aufrechter Position an den Kreislaufstatus des Patienten angepasst werden kann, wurden entsprechende Antriebs- und Lagerungsvorrichtungen für die Beine integriert. Somit war es möglich, die Beine in einer dem physiologischen Gehen entsprechenden Art und Weise (Gelenkwinkel, phasenrichtige Be-/Entlastung der Fußsohle, Geschwindigkeit) zu bewegen. Diese Art der Bewegung stellt nach neuesten neurophysiologischen Erkenntnissen einen maximalen Trigger für die Aktivierung des spinalen Gangmustergenerators im Rückenmark und damit für die Muskelaktivierung an den unteren Extremitäten dar ([7]). Das von uns verwendete Gerät Erigo ist dabei eine Weiterentwicklung des Reha-Steppers.

(Das Bild wurde für die Veröffentlichung entfernt.)

Abbildung 1: Erigo

5.2 Wirkungen (therapeutisch, diagnostisch)

Zu untersuchende Wirkungen:

- Kreislaufstabilisierung – durch eine Verbesserung des venösen Blutrückstromes zum Herzen durch passive Bewegung der Beine und Auslösen von Muskelaktivität infolge einer Triggerung des spinalen Gangprogrammes
- Vigilanzverbesserung – durch simultane Anwendung zweier Aktivierungsformen aus der Komastimulation

Zusätzliche erwartete Therapieeffekte auf Sekundärfolgen einer langen Immobilisation in Folge einer schweren quantitativen Bewusstseinsstörung:

- Osteoporose-Prophylaxe – durch phasenrichtiges Be-/Entlasten von Knochenstrukturen
- Thrombose-Prophylaxe – durch passive Bewegung der Beine und Aufrichtung des Körpers
- Pneumonie-Prophylaxe – durch Erweiterung des intrathorakalen Gasvolumens durch Senkung des Zwerchfells während der Aufrichtung
- Kontrakturen-Prophylaxe – durch passives Durchbewegen der Gelenke mit angepaßter Geschwindigkeit
- Spastikmindernder Effekt – durch passives, reziprokes Durchbewegen und Aktivierung des spinalen Gangprogrammes

5.3 Unerwünschte Wirkungen, sonstige Risiken, Belastungen für den Studienteilnehmer

- Unter der Therapie auf dem Erigo kann es unter ungünstigen Umständen zu Synkopen mit den möglichen Risiken wie Bewusstlosigkeit, anhaltende Bradykardie bzw. Hypotonie kommen. Allerdings ist darauf hinzuweisen, dass diese im Extremfall auftretenden, orthostatischen Synkopen bei den Patienten in der Übergangsphase zwischen Liegen und Aufrichtung auch während der ohnehin stattfindenden Rollstuhlmobilisierung oder der alternativen Stehbretttherapie vorkommen können. Durch die zeitgleiche passive Beinbewegung auf dem Erigo mit vermehrtem Rücktransport des venösen Blutes zum Herzen ist nach derzeitiger Studienlage das Synkopenrisiko auf dem Erigo geringer als auf dem Stehbrett ([6]).
- Die sonstigen Risiken entsprechen denen einer typischen physiotherapeutischen Behandlung, sind somit in Studien- und Kontrollgruppe identisch und werden durch die Studienteilnahme im Vergleich zum üblichen Therapieangebot nicht verändert.

6 Studiendesign(-typ)

Bei der Pilotstudie handelt es sich um eine prospektive, monozentrische, randomisierte Studie im Cross-over Design. Jeder der 20 Studienteilnehmer erhält eine Therapieeinheit auf dem Erigo und eine auf dem Stehbrett. Der Abstand zwischen den Therapieeinheiten beträgt mindestens einen, maximal sieben Tage. Die Reihenfolge der Behandlungsart erfolgt randomisiert.

Die Hauptstudie wird prospektiv, monozentrisch, kontrolliert und randomisiert durchgeführt, zudem wird das Hauptzielkriterium – die Vigilanzverbesserung gemessen an der Coma Recovery Scale – durch einen verblindeten Untersucher erhoben. Die Auswertung der Ergebnisse erfolgt durch eine erfahrene untersuchungsverblindete Untersucherin aus der Neurologischen Klinik Bad Aibling. Diese wird die Daten nach Eingabe in eine Datenbank mit pseudonymisierter Zuordnung zu den Interventionsgruppen auswerten.
Die Patienten erhalten entsprechend der Gruppenzuteilung vier Therapieeinheiten / Woche auf dem Erigo bzw. dem Stehbrett, die Studie ist über drei Wochen vorgesehen. Ungeachtet dessen wird das in der neurologischen Klinik Bad Aibling für apallische und minimal responsive Patienten vorgesehene Therapieangebot aus aktivierender Pflege, Physio- / Ergo- und Schlucktherapie sowie Physikalischer Therapie durchgeführt. Im Bereich Physiotherapie erhalten alle Studienpatienten neben den Erigo – bzw. Stehbrettherapien mindestens fünf weitere Einheiten Einzeltherapie.
Eine Nachuntersuchung ist 3 Wochen nach Therapieende vorgesehen, in den drei Wochen zwischen Therapieende und Nachuntersuchung soll die Mobilisation durch klassische Physiotherapie und nicht auf Erigo oder Kipptisch durchgeführt werden.
.

6.1 Randomisierungsverfahren/ggf. –plan

6.1.1 Pilotstudie

Randomisierungsverfahren: Alle Studienteilnehmer erhalten jeweils eine Therapieeinheit auf dem Erigo und auf dem Stehbrett. Die Reihenfolge der Therapien wird randomisiert. Die Randomisierung erfolgt ex ante in zwei Behandlungsgruppen (1 = Stehbrett - Erigo, 2 = Erigo - Stehbrett) mittels einer computergenerierten Zufallszahlenliste.
Concealment: Die Erstellung der Randomisierungsliste erfolgt durch die Abteilung für medizinische Datenauswertung der Schön-Kliniken.
Ablauf: Jeder Patient, der die Ein- und Ausschlusskriterien erfüllt, wird dem Statistiker oder seinem Vertreter umgehend konsekutiv gemeldet. Dieser teilt den Projektbeteiligten für jeden konsekutiv anfallenden Probanden nach telefonischer oder persönlicher Bekanntgabe der

Patientendaten die Gruppenzugehörigkeit in der festgelegten Reihenfolge (absteigend) mit und notiert auf der Randomisierungsliste Name, Vorname und Geburtsdatum des Probanden. Der erste Patient erhält die Zuteilung mit der Nummer 1, der zweite Patient diejenige mit der Nummer 2 usw. Der anfragende Projektbeteiligte notiert umgekehrt die Gruppenzugehörigkeit. Über die Zuordnungen wird ein Logbuch geführt, das nach Abschluss der Studie zur Einsichtnahme zur Verfügung steht. Somit ist die Geheimhaltung der noch nicht verwendeten Randomisierungsreihenfolge gewährleistet. Ein Drop-out wird durch den zeitlich nach dem Ausscheiden nächstanfallenden einzubeziehenden Probanden unter Beibehaltung der Zuordnung zu Therapie- und Kontrollgruppe ersetzt.

6.1.2 Hauptstudie

Randomisierungsverfahren: Die Studienteilnehmer werden zwei Behandlungsgruppen zugeteilt, Gruppe 1 = Behandlung mit dem Stehbrett, Gruppe 2 = Behandlung mit dem Erigo. Die Gruppenzugehörigkeit wird dabei stratifiziert randomisiert. Die Stratifizierung erfolgt nach den folgenden zwei Kriterien, von denen eine Auswirkung auf das Outcome der Patienten erwartet wird: ursächliche Diagnose (traumatisch versus nicht-traumatisch) und Schwere der Bewusstseinsstörung entsprechend der CRS bei Studienbeginn (apallisch versus minimal responsiv) ([9]).
Concealment und Ablauf erfolgen wie in der Pilotstudie.

6.2 Statistisches Design

6.2.1 Pilotstudie

Entsprechend der statistischen Empfehlung wird das relative Risiko einer Synkope auf Erigo oder Stehbrett sowie die Odds Ratio berechnet.

6.2.2 Hauptstudie

In die Hauptstudie werden insgesamt 50 Patienten aufgenommen.
Die Fallzahlschätzung wurde über das Hauptzielkriterium CRS vorgenommen und basierte auf einer 6-wöchigen Verlaufskontrolle zur Wirkung der Standardtherapie bei 12 minimal responsiven/apallischen Patienten. Für die Differenzwerte der CRS vor und nach einer 6-wöchigen Standardtherapiephase ergab sich eine Verbesserung im Mittelwert von 6,5 Punkten mit einer Varianz von 12,3 (Standardabweichung 3,5). Mit der hier geplanten Studie soll eine zusätzliche Verbesserung der KRS um 3 Punkte erzielt werden. D.h., dass durchschnittlich eine zusätzliche Verbesserung in der Hälfte der zu bewertenden Items der CRS erwartet wird.
Die Fallzahlabschätzung erfolgte für die inferenzstatistische Überprüfung der Hypothesen für ein intervallskaliertes Merkmal ([10]) bei Erfüllung entsprechender Normalverteilungsvoraussetzung. Für α = 5% zweiseitig, Power = 80% ergibt sich bei einer erwarteten Steigerung der CRS von 3 Punkten bei zwei Gruppen eine Gruppengrösse von jeweils 22 Patienten. Bei einer Varianz der Differenzwerte (vor/nach Behandlung) der CRS von $3{,}5^2 = 12{,}3$ kann bei einer Gruppenstärke von n=22 mit einer Irrtumswahrscheinlichkeit von 0,05 und einer Power von 0,8 eine Differenz von mindestens 3 Punkten zwischen den 2 Gruppen als signifikant nachgewiesen werden.
Zusätzlich wurde die Zahl der Therapieabbrecher so berücksichtigt, daß die erforderliche Patientenzahl auch nach Abzug einer erwarteten Drop out-Rate von 10 % erreicht wird. Insgesamt wurde somit eine Gruppengröße von 25 Patienten in das Randomisierungsverfahren einbezogen.

Die statistische Auswertung des Hauptzielkriteriums CRS erfolgt mit einer 2-faktoriellen Varianzanalyse. Sollte die Voraussetzungsprüfung Varianzinhomogenität oder eine nicht normalverteilte Stichprobe erbringen, so werden ersatzweise nonparametrische Verfahren verwendet.

Bei Rücktritt eines Patienten aus der Studie auf Wunsch des Betreuers wird der Patient als Intention-to-treat weitergeführt.

6.3 Einschlußkriterien

- Zustand nach Schädelhirntrauma, hypoxischer Hirnschädigung, intracerebraler Blutung oder ischämischem Hirninfarkt.
- Apallisches Syndrom / minimal responsiver Status entsprechend der Coma Recovery Scale-Revised.

  Für die Hauptstudie erfolgt dabei bei Patienten mit hypoxischer Hirnschädigung eine Beschränkung auf minimal responsive Patienten, da in verschiedenen Studien die schlechte Prognose von apallischen Patienten nach hypoxischer Hirnschädigung gezeigt wurde ([11]) und somit der Einschluss in eine Therapiestudie nicht zielführend erscheint.
- Das Akutereignis liegt nicht länger als 6 Monate zurück.
- Der Patient ist bislang weniger als 30 Minuten in den Stand mobilisiert.
- Der Patient ist nicht jünger als 18 und nicht älter als 70 Jahre alt.

6.4 Ausschlußkriterien

- Akute Herz-Lungen-Erkrankungen wie Pneumonie, Asthma bronchiale, Herzinfarkt vor weniger als 4 Wochen, etc.
- Instabile Brüche jeglicher Art, offene Verletzungen und Decubiti an Rumpf und unteren Extremitäten.
- Ausgeprägte Osteoporose an den unteren Extremitäten.
- Ausgeprägte Kontrakturen an Hüft-, und Kniegelenken.
- Extreme Spastik.
- Herzschrittmacher.
- Schwangerschaft.
- Beinlänge (Ferse – Trochanter major) > 100 cm oder < 70 cm.

7 Studienablauf

7.1 Versuchsdurchführung

Erigo	Stehbrett
Anlage von EKG-Elektroden, Blutdruckmanschette und Pulsoxymeter zur permanenten Messung von Puls, EKG, Blutdruck und Sauerstoffsättigung sowie Durchführung der ersten Messung.	
Patient wird vom Bett/Rollstuhl auf Erigo, bzw. Stehbrett transferiert, fixiert und mittels Bauchgurt gesichert.	
Einstellung der Fußplatten individuell entsprechend der Beinlänge des Patienten.	
Einstellung des Bewegungsausmaßes der Beine auf den bei diesem Patienten maximal möglichen Bereich.	

Starten des Beinantriebes bei weiterhin waagerechter Lage mit maximal tolerierter Geschwindigkeit (Zielwert 40 Schritte/min, mindestens jedoch 20 Schritte/min).	
Es erfolgt die Aufrichtung auf 30°, diese Bedingungen werden für etwa 5 Minuten beibehalten.	
Danach wird der Patient (ggf. unter Beibehaltung der Antriebsparameter) auf 50° aufgerichtet, bei guter Kreislaufsituation schließt sich die Aufrichtung um 70˚ gegenüber der Horizontalen an.	
Mind. 15 Minuten, max. 30 Minuten Therapie unter Beibehaltung des erreichten Aufstellwinkels. Dabei wird die Länge der Mobilisation im vorgegebenen Zeitrahmen möglichst lang gewählt, so lange keine Kreislaufdepression entsprechend der vorgegebenen Kriterien (siehe unten) auftritt.	
Zum Ende der Therapie werden Erigo, bzw. Stehbrett, mit einer kurzen Gewöhnungspause bei etwa 15° wieder auf die waagerechte Lage abgesenkt. Nach der Abschlussmessung Abbau der Messutensilien und Transfer des Patienten zurück in Bett oder Rollstuhl.	

7.2 Voruntersuchungen

Es wird vor Studieneintritt die Coma Recovery Scale erhoben. Zudem wird ein EKG durchgeführt, sollte es nicht bereits während des bisherigen stationären Aufenthaltes durchgeführt worden sein. Weitere oder invasive Untersuchungsmethoden sind nicht geplant.

7.3 Invasive Untersuchungen

Invasive Untersuchungen allein zu Studienzwecken sind nicht vorgesehen.

7.4 Nichtinvasive Untersuchungen in der Pilotstudie

Untersuchungsart:	Zeitpunkt:	Zeitraum:	Häufigkeit:
Aufnahmeuntersuchung, Prüfung der vorliegenden Krankenakte	Anfang	1 Woche	1x
EKG	Anfang	1 Woche	1x
EKG-Monitoring	bei jeder Therapie	1 Woche	2x
Herzfrequenz	bei jeder Therapie	1 Woche	2x
Blutdruck	bei jeder Therapie	1 Woche	2x
Sauerstoffsättigung	bei jeder Therapie	1 Woche	2x
Oberflächen-EMG	Bei jeder Therapie	1 Woche	2x
Hautwiderstand	Vor und nach jeder Therapie	1 Woche	3x
Herzfrequenzvarianz-analyse	Vor und nach jeder Therapie	1 Woche	3x
Ashworth-Skala	Vor und nach jeder Therapie	1 Woche	2x
Coma Recovery Scale-Revised	Vor, während und nach jeder Therapie	1 Woche	6x
Glasgow Coma Scale	Anfang	1 Woche	1x

7.5 Nichtinvasive Untersuchungen in der Hauptstudie

Untersuchungsart:	Zeitpunkt:	Zeitraum:	Häufigkeit:
Aufnahmeuntersuchung, Prüfung der vorliegenden Krankenakte	Anfang	6 Wochen	1x
EKG	Anfang	6 Wochen	1x
EKG-Monitoring	bei jeder Therapie	6 Wochen	9x
Herzfrequenz	bei jeder Therapie	6 Wochen	9x
Blutdruck	bei jeder Therapie	6 Wochen	9x
Sauerstoffsättigung	bei jeder Therapie	6 Wochen	9x
Hautwiderstand	Vor Therapiebeginn, sowie 3 und 6 Wochen nach Therapiebeginn	6 Wochen	3x
Herzfrequenzvarianz-analyse	Vor Therapiebeginn, sowie 3 und 6 Wochen nach Therapiebeginn	6 Wochen	3x
Ashworth-Skala	Vor Therapiebeginn, sowie 3 und 6 Wochen nach Therapiebeginn	6 Wochen	3x
Coma Recovery Scale-R	Vor Therapiebeginn, sowie 3 und 6 Wochen nach Therapiebeginn	6 Wochen	3x
Glasgow-Coma-Skala	Vor Therapiebeginn	6 Wochen	1x
Glasgow-Outcome-Skala	Bei Entlassung aus der stationären Behandlung		1x

8 Medikamentöse Begleittherapie

Medikamentöse Therapien von internistischen, urologischen oder sonstigen Begleiterkrankungen sind bis auf die in den Ausschlusskriterien genannten Bedingungen (akute Pneumonie, Asthma bronchiale) in vollem Umfang erlaubt. Bei interkurrenten Erkrankungen werden begründete Einzelfallentscheidungen getroffen, die im Studienprotokoll vermerkt werden und deren Interpretation dann kommentiert wird.
Eine medikamentöse Therapie mit dem Ziel der Vigilanzsteigerung soll während der Studiendauer nicht angesetzt werden. Sollte eine solche Therapie bereits vor Aufnahme in unsere Klinik, bzw. vor Aufnahme in die Studie bestehen, soll während der Studiendauer keine Veränderung an der Medikation vorgenommen werden. Da trotz guter theoretischer Modelle die momentan vorliegenden klinischen Studien keinen ausreichenden Beweis für den Erfolg einer medikamentösen Vigilanzsteigerung liefern ([8]), ist es ethisch gut vertretbar, den Versuch einer medikamentösen Vigilanzsteigerung auf die Zeit nach Studienende zu verschieben.

9 Definition unerwünschter Ereignisse

(unerwünschte Ereignisse, UE, adverse events, AE) incl. schwerwiegender unerwünschter Ereignisse (SUE; serious adverse events, SAE).

9.1 Definition eines Unerwünschten Ereignisses (UE/AE)

Ein UE ist jedes ungünstige und unerwartete Ereignis (einschließlich z.B. abnormaler Laborwerte), jedes Symptom oder jede Erkrankung, die in zeitlichem Zusammenhang mit dem Gebrauch eines Medizinproduktes auftritt, unabhängig davon, ob ein Zusammenhang mit dem Medizinprodukt angenommen wird oder nicht. Vor allem sollte jede Veränderung von Laborwerten oder vitalen Funktionen, die bei dem betreffenden Patienten zum Absetzen der Studienanwendung, als UE angesehen werden.

9.2 Definition eines Schwerwiegenden Unerwünschten Ereignisses (SUE/SAE)

Ein unerwünschtes Ereignis wird als schwerwiegend eingeschätzt (modifiziert nach *The Trial Investigator`s GCP Handbook 1997*), wenn der Patient:

- stirbt.
- Ein lebensbedrohendes Ereignis eintritt (die Lebensgefahr muss tatsächlich bestanden haben; es reicht nicht, dass das Ereignis, wäre es schwerer gewesen, zum Tode hätte führen können).
- ein bestehender Krankenhausaufenthalt verlängert werden muß.
- eine dauerhafte oder entscheidende Behinderung erfährt.
- Nachkommen mit einer kongenitalen Anomalie oder einem Geburtsdefekt bekommt.
- ein anderes, nach medizinischer Einschätzung klinisch relevantes Ereignis erleidet.

9.3 Dokumentation und Meldung

9.3.1 Dokumentation eines unerwünschten Ereignisses:

Jedes unerwünschte Ereignis muss im Bericht für unerwünschte Ereignisse dokumentiert werden.

In jedem Fall muss der Prüfarzt im UE-Bericht eindeutig identifizierbar sein. Der Bericht muss vom Prüfarzt datiert und unterzeichnet sein. Er sollte außerdem eine Bewertung des Kausalzusammenhanges zwischen Ereignis und Medizinprodukt enthalten.
Die Beschreibung des unerwünschten Ereignisses enthält den Zeitpunkt des Auftretens, die Dauer, Schwere, Intensität, Ausgang und die Beziehung zur Studienanwendung und die notwendige Behandlung. Der Prüfarzt soll die Intensität jedes unerwünschten Ereignisses, basierend auf der folgenden Einteilung, einschätzen:

Intensität:

- **Leicht:** Das unerwünschte Ereignis ist vorübergehend und vom Patienten leicht zu ertragen.
- **Mäßig:** Das unerwünschte Ereignis bereitet dem Patienten Unannehmlichkeiten und behindert ihn bei seinen üblichen Tätigkeiten.
- **Schwer:** Das unerwünschte Ereignis bereitet dem Patienten erhebliche Störungen seiner üblichen Aktivitäten.

Therapeutische Konsequenzen müssen dokumentiert werden, z.B. medikamentöse Behandlung, Ausschluss aus der Studie o.ä.

Als ein „unerwartetes unerwünschtes Ereignis“ im Zusammenhang mit dem Prüfprodukt (MP) bezeichnet man ein Ereignis, dessen Natur oder Schweregrad nicht mit den bekannten Daten über die Sicherheit und Verträglichkeit des Produkts übereinstimmen.

Kausalität: Für die Beurteilung des Zusammenhangs zwischen der Anwendung des Prüfproduktes (MP) und dem UE werden folgende Definitionen verwendet:

- **Sicher:** Eine Reaktion, die einem nachvollziehbaren zeitlichen Ablauf nach der Anwendung des Prüfproduktes folgt, einem bekannten oder erwarteten Antwortmuster auf das verdächtigte Prüfprodukt folgt und nach Absetzen oder Dosisreduktion verschwindet und bei erneuter Exposition wieder auftritt.

- **Wahrscheinlich:** Eine Reaktion, die einem nachvollziehbaren zeitlichen Ablauf nach der Anwendung des Prüfproduktes folgt, einem bekannten oder erwarteten Antwortmuster auf das verdächtigte Prüfprodukt folgt und nach Absetzen oder Dosisreduktion verschwindet und nicht durch die bekannten Merkmale des klinischen Zustandes des Patienten erklärt werden kann.

- **Möglich:** Eine Reaktion, die einem nachvollziehbaren zeitlichen Ablauf nach der Anwendung des Prüfproduktes folgt, einem bekannten oder erwarteten Antwortmuster auf das verdächtigte

Prüfprodukt folgt, die aber leicht auch durch eine Reihe anderer Faktoren hervorgerufen worden sein könnte.

- **Ohne Zusammenhang:** Eine Reaktion, bei der ausreichend Informationen vorliegen für die Annahme, dass kein Zusammenhang mit dem Prüfprodukt besteht.

- **Nicht beurteilbar:** Eine Einschätzung des Zusammenhangs ist nicht möglich.

9.3.2 Meldung und Dokumentation eines Schwerwiegenden Unerwünschten Ereignisses

Sobald während der Studie einschließlich der Einschlussphase ein Schwerwiegendes unerwünschtes Ereignis beobachtet wird, erstellt der Prüfarzt einen UE-Bericht. In diesem muss der Prüfarzt eindeutig identifizierbar sein. Der Bericht muss vom Prüfarzt datiert und unterzeichnet sein. Er sollte außerdem eine Bewertung des Kausalzusammenhanges zwischen Ereignis und Medizinprodukt enthalten.
Eine Unterbrechung der Studienteilnahme wird ebenfalls sofort dokumentiert.

9.4 Procedere während und nach der Studie

Der Prüfarzt verfolgt die Entwicklung von unerwünschten klinischen Ereignissen oder krankhaften Laborwerten bis zur Heilung oder Stabilisierung des Zustandes des Studienteilnehmers.

10 Abbruchkriterien

Individuell

- Bei Absinken des Blutdrucks um 20 mmHg systolisch oder um mehr als 10 mmHg diastolisch während der Aufrichtungsphase wird auf den nächstniedrigeren tolerierten Aufrichtwinkel 30° oder 50° zurückgekehrt und die Therapie mit diesem Aufrichtwinkel fortgesetzt.
- Gleicheres Procedere erfolgt bei Symptomen einer Präsynkope (Tachypnoe, Blässe, vermehrtes Schwitzen, ...).
- Bei Absinken des Pulses unter 40 Schläge/Min. bzw. Überschreiten von 140 Schlägen/Min. wird die Therapie sofort abgebrochen.
- Ebenso bei arterieller Hypotonie (<80 mmHg).
- Auftreten einer neuen Erkrankung oder Komplikation der Grunderkrankung, die eine Fortführung der Studie unmöglich macht.
- Der gesetzliche Betreuer wünscht den Abbruch der Studienteilnahme.

11 Ethische und rechtliche Aspekte

11.1 Ethische Grundlagen

Die klinische Prüfung wird in Übereinstimmung mit der Deklaration von Helsinki in der aktuellen Fassung von 1996 und unter Beachtung der ICH-GCP Richtlinien durchgeführt.

Die Teilnahme der Patienten/Probanden an der Untersuchung ist freiwillig; die Zustimmung kann jederzeit, ohne Angabe von Gründen und ohne Nachteile für die weitere medizinische Versorgung, zurückgezogen werden.

11.1.1 Patienten-/Probandeninformation/Einverständniserklärung

Da die Studie die Wirksamkeit eines Therapieverfahren der frühen neurologischen Rehabilitation evaluiert, ist ein Einschlusskriterium die schwere Hirnschädigung mit schwerer quantitativer

Bewusstseinsstörung (apallisches Syndrom oder minimal responsiver Status). Folglich handelt es sich ausschließlich um Patienten, die – außer bei Vorliegen einer Vorsorgevollmacht - eine gesetzliche Betreuung benötigen.
Im Rahmen dieser Studie werden nur Therapieverfahren bzw. eine Kombination von Therapieverfahren eingesetzt (Vertikalisation und passive Bewegung), die bereits seit Jahrzehnten in der neurologischen Frührehabilitation angewandt werden. Zu diesen Verfahren gibt es zwar keine ausreichende Datenlage aus kontrollierten klinischen Studien bezüglich der Wirksamkeit, aufgrund der langen klinischen Erfahrung mit diesen Verfahren ist jedoch nach unserer Einschätzung das Vorenthalten, nicht die Durchführung dieser Stimulationsmethoden an Patienten ethisch zweifelhaft. Zudem ist durch die neue Therapie auf dem Erigo das Risiko von Synkopen als unerwünschte Begleiterscheinung - wie oben dargelegt - eher geringer als mit der klassischen Stehbretttherapie. Insgesamt ist somit durch den Erigo im Vergleich zum etablierten Stehbrett eine Konstanz oder Verbesserung in der Wirksamkeit bei Konstanz oder Reduktion der Risiken auszugehen.
Die gesetzlichen Betreuer der Patienten werden vor Studienbeginn schriftlich und mündlich über Wesen und Tragweite der geplanten Untersuchung, insbesondere über den möglichen Nutzen für die Gesundheit der Patienten und eventuelle Risiken, aufgeklärt. Ihre Zustimmung wird durch Unterschrift auf der Einwilligungserklärung dokumentiert.

11.2 Rechtliche Grundlagen: Einhaltung der Vorschriften des MPG, DSG

Die Bestimmungen des Medizinproduktegesetzes (MPG) werden eingehalten und die "Grundsätze für die ordnungsgemäße Durchführung klinischer Prüfung von Arzneimitteln" sowie das Bundesdatenschutzgesetz (BDSG) finden Anwendung.

11.2.1 Meldung der Prüfung

Gemäß § 17 MPG wird die Studie unter Benennung des Leiters der klinischen Prüfung bei der zuständigen Landesbehörde (Regierungspräsidium) gemeldet.

11.2.2 Votum der Ethikkommission

Der Prüfplan wird vor Studienbeginn der Ethikkommission der Landesärztekammer Bayern zur Begutachtung vorgelegt. Es wird nicht mit dem Einschluss von Probanden/Patienten begonnen, bevor nicht das schriftliche Votum der Ethikkommission vorliegt.

11.2.3 Patienten-Versicherung

Eine zusätzliche Versicherung der Patienten ist nicht notwendig, da nur zugelassene Medizinprodukte verwendet werden. Die geplanten Untersuchungen sind zudem nicht invasiv, es werden im Vergleich zur Standardtherapie keine erweiterten Risiken erwartet.
Die Neurologische Klinik Bad Aibling verfügt über eine Haushaftpflichtversicherung (Versicherungsgesellschaft: Bayerischer Versicherungsverband, Maximilianstr. 53, 80530 München, Versicherungsschein-Nr.: HV 77281 0500), mit der alle diagnostischen und therapeutischen Maßnahmen abgedeckt sind. Die Versicherungssumme für Personenschäden beläuft sich auf 5.200.000 Euro.

11.2.4 Datenschutz/Einblick in Originalkrankenunterlagen

Die Namen der Patienten und alle anderen vertraulichen Informationen unterliegen der ärztlichen Schweigepflicht und den Bestimmungen des Bundesdatenschutzgesetzes. Eine Weitergabe von Patientendaten erfolgt ggf. nur in anonymisierter Form. Sollte es im Verlauf der Studie erforderlich werden, den Namen eines Patienten zu identifizieren (Monitoring), so ist dies möglich, da die Patienten in der schriftlichen Einwilligung erklärt haben, dass sie den Prüfarzt von seiner Schweigepflicht insofern entbinden, als staatliche Gesundheitsbehörden Einsicht in ihre Originalkrankenunterlagen nehmen dürfen, um den ordnungsgemäßen Ablauf der Studie zu überprüfen.

12 Liste der verwendeten Medizinprodukte

In der Therapie verwendet wird der Erigo der Firma Hocoma, ein nach MPG zugelassenes Medizinprodukt im Rahmen der zugelassenen Funktion (CE 0124). Als Stehbrett sowie als Geräte für die o.g. geplanten Untersuchungen werden ausschließlich Medizinprodukte im Rahmen ihrer zugelassenen Funktion verwendet, die im üblichen Klinikbetrieb der Neurologischen Klinik Bad Aibling verwendet werden.

13 Unterschriften

Studienverantwortlicher Chefarzt Dr. med. Dipl.-Psych. F. Müller ____________________________

Studienverantwortliche Ärztin Dr. med. M. Luther ______________________________

Projektleiter Prof. Dr. med. E. Koenig ______________________________

14 Literatur

[1] Lombardi F, Taricco M, De Tanti A, Telaro E, Liberati A, Sensory stimulation of brain-injured individuals in coma or vegetative state: results of a Cochrane systematic review. Clin Rehabil. 2002;16:464-72.

[2] Lippert-Gruner M, Wedekind C, Ernestus RI, Klug N: Early rehabilitative concepts in therapy of the comatose brain injured patients. Acta Neurochir Suppl. 2002;79:21-3.

[3] Welch R. Tilt-table therapy in rehabilitation of the trauma patient with brain damage and spinal injury. Nurs Clin North Am 1970; 5:621-30.

[4] Muraki S, Ehara Y, Yamasaki M: Cardiovascular responses at the onset of passive leg cycle exercise in paraplegics with spinal cord injury. Eur J Appl Physiol 2000; 81:271-74.

[5] Faghri PD, Yount JP, Pesce WJ, Seetharama S, Votto JJ., Circulatory hypokinesis and functional electric stimulation during standing in persons with spinal cord injury. Arch Phys Med Rehabil 2001; 82:1587-95.

[6] Czell D, Schreier R, Rupp R, Eberhard S, Colombo G, Dietz V: Influence of passive leg movements on blood circulation on the tilt table in healthy adults. Neuroengineering Rehabil. 2004 Oct 25;1(1):4.

[7] Duysens J., Van de Crommert H, Neural Control of locomotion: Part 1: The central pattern generator from cats to humans. Gait & Posture 1998; 7:131-141.

[8] Richer E, Tell L: Indications, efficacy and tolerance of drug therapy in view of improving recovery of consciousness following a traumatic brain injury. Ann Readapt Med Phys. 2003; 46:177-83.

[9] The Multi-Society Task Force on PVS: Medical Aspects of the Persistent Vegetative State (First of Two Parts). N Engl J Med 1994; 330:1499-508.

[10] Kalmar K, Giacino J. The JFK Coma Recovery Scale: An Ordinal or Interval Measure? Arch Phys Med Rehabil 2000; 81:1619.

[11] The Multi-Society Task Force on PVS: Medical Aspects of the Persistent Vegetative State (Second of Two Parts). N Engl J Med 1994; 330:1572-9.

[12]) Giacino J, Kalmar K, Whyte J. The JFK Coma Recovery Scale-Revised: Measurement Characteristics and Diagnostic Utility. Arch Phys Med Rehabil 2004; 85:2020-9.
