# Supplementary material for: Tilt Table Therapies for Patients with Severe Disorders of Consciousness: A Randomized, Controlled Trial
Source: PLoS One. 2015 Dec 1;10(12):e0143180. doi: 10.1371/journal.pone.0143180 (PMC4666666; doi:10.1371/journal.pone.0143180)
Supplement: S1 Table — Abbr.: TT, tilt table; f, female; m, male; TBI, traumatic brain injury; CRS-R, coma recovery scale-revised; VS, vegetative state; MCS, minimally conscious state; GCS, Glasgow coma scale. (DOCX) [file pone.0143180.s006.docx]

**S1_Table: Individual patient characteristics**

| Patient | Intervention | Sex | Type of injury | Category of injury | CRS-R at randomization | Age [years] | Time from injury to randomization [weeks] | GCS at study onset |
| --- | --- | --- | --- | --- | --- | --- | --- | --- |
| 1 | Erigo | m | stroke | non-traumatic | VS | 47 | 8 | 9 |
| 2 | Erigo | m | TBI | traumatic | MCS | 41 | 5 | 11 |
| 4 | Tilt table | m | stroke | non-traumatic | VS | 23 | 5 | 8 |
| 5 | Tilt table | f | TBI | traumatic | VS | 54 | 11 | 10 |
| 6 | Tilt table | f | TBI | traumatic | MCS | 40 | 5 | 10 |
| 7 | Erigo | f | stroke | non-traumatic | MCS | 46 | 6 | 9 |
| 8 | Erigo | m | TBI | traumatic | MCS | 57 | 14 | 11 |
| 9 | Erigo | f | TBI | traumatic | VS | 23 | 4 | 9 |
| 10 | Tilt table | f | stroke | non-traumatic | VS | 71 | 6 | 4 |
| 12 | Tilt table | m | stroke | non-traumatic | MCS | 56 | 9 | 9 |
| 13 | Tilt table | m | hypoxia | non-traumatic | MCS | 60 | 10 | 8 |
| 14 | Tilt table | m | stroke | non-traumatic | MCS | 53 | 10 | 11 |
| 16 | Erigo | m | stroke | non-traumatic | MCS | 74 | 8 | 9 |
| 17 | Erigo | m | hypoxia | non-traumatic | MCS | 54 | 6 | 10 |
| 18 | Erigo | m | stroke | non-traumatic | VS | 43 | 4 | 9 |
| 19 | Tilt table | m | stroke | non-traumatic | MCS | 68 | 10 | 10 |
| 20 | Tilt table | m | hypoxia | non-traumatic | MCS | 60 | 7 | 9 |
| 21 | Erigo | f | stroke | non-traumatic | MCS | 59 | 8 | 7 |
| 22 | Erigo | m | hypoxia | non-traumatic | VS | 64 | 10 | 9 |
| 23 | Erigo | f | TBI | traumatic | MCS | 26 | 4 | 9 |
| 25 | Tilt table | m | hypoxia | non-traumatic | MCS | 32 | 9 | 12 |
| 26 | Tilt table | m | stroke | non-traumatic | VS | 60 | 7 | 9 |
| 27 | Erigo | m | hypoxia | non-traumatic | MCS | 25 | 4 | 11 |
| 28 | Tilt table | f | stroke | non-traumatic | VS | 67 | 9 | 10 |
| 29 | Tilt table | m | stroke | non-traumatic | MCS | 42 | 5 | 11 |
| 30 | Erigo | f | stroke | non-traumatic | MCS | 63 | 8 | 4 |
| 31 | Tilt table | f | stroke | non-traumatic | MCS | 68 | 9 | 7 |
| 32 | Erigo | m | stroke | non-traumatic | MCS | 51 | 7 | 9 |
| 33 | Tilt table | f | hypoxia | non-traumatic | MCS | 43 | 8 | 9 |
| 34 | Erigo | f | stroke | non-traumatic | VS | 49 | 7 | 3 |
| 36 | Erigo | m | stroke | non-traumatic | MCS | 68 | 6 | 8 |
| 37 | Tilt table | m | stroke | non-traumatic | MCS | 56 | 5 | 8 |
| 38 | Tilt table | m | hypoxia | non-traumatic | MCS | 36 | 6 | 12 |
| 39 | Tilt table | f | stroke | non-traumatic | MCS | 53 | 7 | 9 |
| 40 | Erigo | f | stroke | non-traumatic | VS | 49 | 13 | 10 |
| 41 | Erigo | f | stroke | non-traumatic | MCS | 74 | 14 | 11 |
| 42 | Erigo | f | stroke | non-traumatic | MCS | 69 | 11 | 13 |
| 43 | Tilt table | m | hypoxia | non-traumatic | MCS | 55 | 4 | 6 |
| 44 | Erigo | f | TBI | traumatic | VS | 51 | 7 | 8 |
| 45 | Tilt table | f | stroke | non-traumatic | MCS | 61 | 14 | 4 |
| 46 | Erigo | m | TBI | traumatic | MCS | 71 | 4 | 10 |
| 47 | Tilt table | m | TBI | traumatic | MCS | 27 | 14 | 9 |
| 49 | Tilt table | m | stroke | non-traumatic | VS | 59 | 11 | 6 |
| 50 | Erigo | m | TBI | traumatic | VS | 57 | 9 | 9 |

Abbr.: TT, tilt table; f, female; m, male; TBI, traumatic brain injury; CRS-R, coma recovery scale-revised; VS, vegetative state; MCS, minimally conscious state; GCS, Glasgow coma scale.
