# Supplementary material for: Tilt Table Therapies for Patients with Severe Disorders of Consciousness: A Randomized, Controlled Trial
Source: PLoS One. 2015 Dec 1;10(12):e0143180. doi: 10.1371/journal.pone.0143180 (PMC4666666; doi:10.1371/journal.pone.0143180)
Supplement: S2 Table — Abbr.: TT, tilt table. *, missing data refilled with the last observation carried forward method. (DOCX) [file pone.0143180.s007.docx]

**S2_Table: Individual CRS-R values**

| Patient | Intervention | Baseline | Week 3 | Week 6 |
| --- | --- | --- | --- | --- |
| 1 | Erigo | 6 | 6 | 8 |
| 2 | Erigo | 17 | 20 | 23 |
| 4 | TT | 12 | 16 | 21 |
| 5 | TT | 7 | 15 | 19 |
| 6 | TT | 13 | 23 | 23 |
| 7 | Erigo | 14 | 16 | 17 |
| 8 | Erigo | 13 | 4 | 4* |
| 9 | Erigo | 7 | 14 | 18 |
| 10 | TT | 3 | 8 | 8* |
| 12 | TT | 9 | 15 | 16 |
| 13 | TT | 8 | 9 | 11 |
| 14 | TT | 13 | 17 | 21 |
| 16 | Erigo | 13 | 12 | 12* |
| 17 | Erigo | 10 | 16 | 13 |
| 18 | Erigo | 14 | 18 | 18 |
| 19 | TT | 10 | 21 | 19 |
| 20 | TT | 13 | 22 | 23 |
| 21 | Erigo | 7 | 10 | 6 |
| 22 | Erigo | 15 | 6 | 6* |
| 23 | Erigo | 11 | 23 | 23 |
| 25 | TT | 15 | 19 | 17 |
| 26 | TT | 14 | 23 | 23 |
| 27 | Erigo | 10 | 10 | 8 |
| 28 | TT | 14 | 17 | 19 |
| 29 | TT | 12 | 20 | 23 |
| 30 | Erigo | 10 | 12 | 17 |
| 31 | TT | 11 | 19 | 20 |
| 32 | Erigo | 10 | 19 | 22 |
| 33 | TT | 12 | 10 | 10* |
| 34 | Erigo | 5 | 8 | 11 |
| 36 | Erigo | 13 | 14 | 18 |
| 37 | TT | 15 | 18 | 22 |
| 38 | TT | 15 | 19 | 23 |
| 39 | TT | 15 | 23 | 23 |
| 40 | Erigo | 8 | 12 | 12 |
| 41 | Erigo | 18 | 16 | 16* |
| 42 | Erigo | 14 | 19 | 21 |
| 43 | TT | 9 | 12 | 19 |
| 44 | Erigo | 12 | 12 | 9 |
| 45 | TT | 9 | 13 | 19 |
| 46 | Erigo | 16 | 21 | 20 |
| 47 | TT | 8 | 9 | 17 |
| 49 | TT | 7 | 9 | 6 |
| 50 | Erigo | 12 | 13 | 13 |

Abbr.: TT, tilt table. *, missing data refilled with the last observation carried forward method.
