# Supplementary material for: Tilt Table Therapies for Patients with Severe Disorders of Consciousness: A Randomized, Controlled Trial
Source: PLoS One. 2015 Dec 1;10(12):e0143180. doi: 10.1371/journal.pone.0143180 (PMC4666666; doi:10.1371/journal.pone.0143180)
Supplement: S4 Table — Abbr.: MD, missing data. (DOCX) [file pone.0143180.s009.docx]

**S4_Table: Individual Ashworth scores - lower extremities**

|  | Ankle extension right | | Ankle flexors right | | Ankle extension left | | Ankle flexors left | | Knee extensors right | | Knee flexors right | | Knee extensors left | | Knee flexors left | | Hip extensors left | | Hip extensors right | | Hip flexors left | | Hip flexors right | |
| --- | --- | --- | --- | --- | --- | --- | --- | --- | --- | --- | --- | --- | --- | --- | --- | --- | --- | --- | --- | --- | --- | --- | --- | --- |
| Patient | Baseline | Week 3 | Baseline | Week 3 | Baseline | Week 3 | Baseline | Week 3 | Baseline | Week 3 | Baseline | Week 3 | Baseline | Week 3 | Baseline | Week 3 | Baseline | Week 3 | Baseline | Week 3 | Baseline | Week 3 | Baseline | Week 3 |
| 1 | 0 | 0 | 1 | 0 | 0 | 0 | 1 | 0 | 0 | 0 | 1+ | 1+ | 0 | 0 | 1+ | 0 | 0 | 0 | 0 | 1+ | 1+ | 1+ | 1+ | 1+ |
| 2 | MD | 0 | MD | 1+ | MD | 0 | MD | 1 | MD | 1+ | MD | 1+ | MD | 0 | MD | 0 | MD | 0 | MD | 1 | MD | 0 | MD | 1 |
| 4 | 0 | 0 | 0 | 0 | 0 | 0 | 1 | 0 | 1 | 0 | 0 | 0 | 0 | 0 | 0 | 0 | 1 | 0 | 0 | 1+ | 0 | 0 | 0 | 0 |
| 5 | 0 | 0 | 0 | 0 | 0 | 0 | 0 | 0 | 0 | 0 | 0 | 0 | 0 | 0 | 0 | 0 | 0 | 0 | 0 | 0 | 0 | 0 | 0 | 0 |
| 6 | 0 | 0 | 0 | 1+ | 0 | 0 | 1 | 1 | 0 | 1 | 0 | 0 | 1 | 1 | 0 | 0 | 0 | 0 | 0 | 0 | 0 | 0 | 0 | 0 |
| 7 | 0 | 0 | 0 | 0 | 0 | 0 | 0 | 0 | 0 | 0 | 0 | 0 | 0 | 0 | 0 | 0 | 0 | 1+ | 0 | 0 | 0 | 0 | 0 | 0 |
| 8 | 0 | MD | 0 | MD | 0 | 0 | 0 | 0 | 0 | 0 | 0 | 0 | 0 | 0 | 0 | 0 | 0 | 0 | 0 | 0 | 0 | 0 | 0 | 0 |
| 9 | MD | 0 | MD | 1 | MD | 0 | MD | 0 | 0 | 0 | 0 | 0 | 0 | 0 | 0 | 0 | 0 | 0 | 0 | 0 | 0 | 0 | 0 | 0 |
| 10 | 0 | MD | 0 | MD | 0 | MD | 0 | MD | 0 | MD | 0 | MD | 0 | MD | 0 | MD | 0 | MD | 0 | MD | 0 | MD | 0 | MD |
| 12 | 0 | 0 | 0 | 0 | MD | MD | MD | MD | 0 | 0 | 0 | 0 | 0 | 0 | 0 | 1 | 0 | 0 | 0 | 1+ | 0 | 0 | 1 | 1+ |
| 13 | 0 | 0 | 0 | 0 | 0 | 0 | 0 | 0 | 0 | 0 | 0 | 0 | 0 | 0 | 0 | 0 | 0 | 0 | 0 | 0 | 0 | 0 | 0 | 0 |
| 14 | 0 | 0 | 0 | 0 | 0 | 0 | 0 | 1 | 0 | 0 | 0 | 0 | 0 | 0 | 1 | 1 | 0 | 1 | 0 | 0 | 1 | 1 | 0 | 0 |
| 16 | 0 | 0 | 1 | 0 | MD | 0 | MD | 0 | 0 | 0 | 2 | 0 | MD | 2 | MD | MD | MD | 2 | 1+ | 1+ | MD | 2 | 0 | 1+ |
| 17 | 0 | 0 | 0 | 0 | 0 | 0 | 0 | 0 | 0 | 0 | 0 | 0 | 0 | 0 | 0 | 0 | 0 | 0 | 0 | 0 | 0 | 0 | 0 | 0 |
| 18 | 3 | 3 | 0 | 3 | 3 | 1 | 0 | 0 | 2 | 3 | 0 | 0 | 0 | 0 | 0 | 0 | 0 | 0 | 0 | 3 | 0 | 0 | 0 | 0 |
| 19 | 0 | 0 | 0 | 1 | 0 | 1 | 0 | 1 | 1+ | 3 | 1+ | 1+ | 0 | 1+ | 0 | 1+ | 0 | 0 | 1 | 1+ | 0 | 0 | 0 | MD |
| 20 | 1 | 0 | 0 | 0 | 1 | 0 | 0 | 0 | 0 | 0 | 0 | 0 | 0 | 0 | 0 | 0 | 0 | 0 | 0 | 0 | 0 | 0 | 0 | 0 |
| 21 | 0 | 0 | 0 | 0 | 0 | 1 | 0 | 0 | 0 | 0 | 0 | 0 | 0 | 0 | 0 | 0 | 0 | 0 | 0 | 0 | 0 | 0 | 0 | 0 |
| 22 | 3 | 2 | 3 | 2 | 0 | 2 | 2 | 2 | 3 | 2 | 2 | 2 | 2 | 2 | 0 | 2 | 2 | 2 | 3 | 2 | 0 | 2 | 2 | 2 |
| 23 | 0 | 0 | 0 | 1 | 0 | 0 | 0 | 1 | 0 | 0 | 0 | 0 | 0 | 0 | 0 | 0 | 0 | 0 | 0 | 0 | 0 | 0 | 0 | 0 |
| 25 | 0 | 0 | 0 | 0 | 0 | 0 | 0 | 0 | MD | 0 | MD | 0 | 0 | 0 | MD | 2 | 0 | 0 | MD | 0 | MD | 2 | MD | 0 |
| 26 | 0 | 0 | 0 | 0 | 0 | 0 | 0 | 0 | 0 | 1 | 0 | 1 | 0 | 0 | 0 | 0 | 0 | 0 | 0 | 2 | 0 | 0 | 0 | 2 |
| 27 | 0 | 0 | 2 | 0 | 0 | 0 | 0 | 0 | 0 | MD | 0 | 3 | 0 | MD | 0 | 3 | 0 | MD | 0 | MD | 0 | 3 | 0 | 3 |
| 28 | 0 | 0 | 0 | 0 | 0 | 0 | 0 | 0 | 0 | 0 | 0 | 0 | 0 | 0 | 0 | 0 | 0 | 0 | 0 | 0 | 0 | 0 | 0 | 0 |
| 29 | 0 | 0 | 0 | 0 | 0 | 0 | 0 | 0 | 0 | 0 | 0 | 0 | 1+ | 0 | 0 | 0 | 1+ | 0 | 0 | 0 | 0 | 0 | 0 | 0 |
| 30 | 0 | MD | 0 | MD | 0 | MD | 0 | MD | 0 | MD | 0 | MD | 0 | MD | 0 | MD | 0 | MD | 0 | MD | 0 | MD | 0 | MD |
| 31 | 0 | MD | 0 | MD | 0 | MD | 0 | MD | 0 | MD | 0 | MD | 0 | MD | 0 | MD | 0 | MD | 0 | MD | 0 | MD | 0 | MD |
| 32 | 0 | 0 | 1+ | 1 | 0 | 0 | 1 | 1 | 0 | 0 | 0 | 0 | 0 | 0 | 0 | 0 | 0 | 0 | 0 | 0 | 0 | 0 | 0 | 0 |
| 33 | 0 | 0 | 0 | 3 | 0 | 0 | 0 | 3 | 0 | 0 | 0 | 0 | 0 | 0 | 0 | 2 | 0 | 0 | 0 | 0 | 0 | 0 | 0 | 0 |
| 34 | 0 | 0 | 0 | 0 | 0 | 0 | 0 | 0 | 0 | 0 | 0 | 0 | 0 | 0 | 0 | 0 | 0 | 0 | 0 | 0 | 0 | 0 | 0 | 0 |
| 36 | 0 | 0 | 0 | 0 | 0 | MD | 0 | MD | 0 | 0 | 0 | 0 | 0 | MD | 0 | MD | 0 | MD | 0 | 0 | 0 | MD | 0 | 0 |
| 37 | MD | 0 | MD | 0 | 0 | 0 | 1+ | 0 | MD | 0 | MD | 0 | 0 | 0 | 0 | 0 | 0 | 0 | MD | 0 | 0 | 0 | MD | 0 |
| 38 | 2 | 1 | 2 | 2 | 2 | 1 | 2 | 2 | 0 | 2 | 0 | 1+ | 0 | 2 | 0 | 1+ | 0 | 1+ | 0 | 2 | 0 | 1+ | 0 | 2 |
| 39 | 0 | 0 | 1 | 0 | 0 | 0 | 0 | 0 | 0 | 0 | 0 | 0 | 0 | 0 | 0 | 0 | 0 | 0 | 0 | MD | 0 | 0 | 0 | 0 |
| 40 | 0 | 0 | 0 | 0 | 0 | 0 | 0 | 0 | 0 | MD | 0 | MD | 0 | 0 | 0 | 0 | 0 | 0 | 0 | MD | 0 | 0 | 0 | MD |
| 41 | MD | 0 | MD | 0 | 0 | 0 | 1 | 1 | MD | 2 | MD | 1+ | 0 | 1 | 0 | 1 | 0 | 1+ | MD | 3 | 0 | 1+ | MD | 2 |
| 42 | 0 | 0 | 0 | 0 | 0 | 0 | 0 | 0 | 0 | 0 | 0 | 0 | 0 | 0 | 0 | 0 | 0 | 0 | 0 | 0 | 0 | 0 | 0 | 0 |
| 43 | 0 | 0 | 0 | 0 | 0 | 0 | 0 | 0 | 0 | 0 | 1+ | 1+ | 0 | 0 | 0 | 1+ | 0 | 1 | 0 | 0 | 0 | 0 | 1 | 0 |
| 44 | 0 | MD | 1 | MD | 0 | 0 | 1 | 0 | 0 | MD | 0 | MD | 0 | 0 | 0 | 0 | 0 | 0 | 1+ | MD | 0 | 0 | 0 | MD |
| 45 | 0 | 0 | 1 | 0 | 0 | 0 | 1 | 3 | 0 | 0 | 0 | 0 | 0 | 0 | 0 | 0 | 0 | 0 | 0 | 0 | 0 | 0 | 0 | 0 |
| 46 | 0 | 2 | 0 | 2 | 0 | 0 | 1 | 0 | 0 | 0 | 0 | 0 | 0 | 0 | 0 | 0 | 0 | 0 | 0 | 0 | 0 | 0 | 0 | 0 |
| 47 | 0 | 1 | 1 | 1 | 0 | 0 | 1 | 1 | 1 | 1 | 0 | 1 | 0 | 0 | 0 | 0 | 1 | 1 | 2 | 3 | 0 | 0 | 1 | 1 |
| 49 | 0 | 0 | 0 | 0 | 0 | 0 | 0 | 0 | 0 | 0 | 0 | 0 | 0 | 0 | 0 | 0 | 0 | 0 | 0 | 0 | 0 | 0 | 0 | 0 |
| 50 | 0 | 0 | 0 | 0 | 0 | 0 | 0 | 0 | 0 | 0 | 0 | 0 | 0 | 2 | 0 | 0 | 0 | 0 | 0 | 0 | 0 | 0 | 0 | 0 |

Abbr.: MD, missing data.
