# Supplementary material for: Tilt Table Therapies for Patients with Severe Disorders of Consciousness: A Randomized, Controlled Trial
Source: PLoS One. 2015 Dec 1;10(12):e0143180. doi: 10.1371/journal.pone.0143180 (PMC4666666; doi:10.1371/journal.pone.0143180)
Supplement: S5 Table — Abbr.: n.a., no therapy applied or not documented; interruption 0, no therapy interruption; interruption 1, therapy interruption necessary. (DOCX) [file pone.0143180.s010.docx]

S5_Table: Therapy documentation

| Intervention | Patient | Therapy session (number) | Verticalization duration in minutes | Maximal verticalization angle | Duration maximal angle in minutes | Interruption |
| --- | --- | --- | --- | --- | --- | --- |
| Erigo | 1 | 1 | 25 | 70 | 13 | 0 |
| Erigo | 1 | 2 | 19 | 70 | 9 | 1 |
| Erigo | 1 | 3 | 22 | 70 | 13 | 0 |
| Erigo | 1 | 4 | 22 | 70 | 6 | 0 |
| Erigo | 1 | 5 | 24 | 70 | 20 | 0 |
| Erigo | 1 | 6 | 28 | 70 | 19 | 0 |
| Erigo | 1 | 7 | 25 | 70 | 14 | 0 |
| Erigo | 1 | 8 | 17 | 70 | 7 | 1 |
| Erigo | 1 | 9 | n.a. | n.a. | n.a. | n.a. |
| Erigo | 1 | 10 | 14 | 70 | 7 | 0 |
| Erigo | 2 | 1 | 27 | 70 | 2 | 0 |
| Erigo | 2 | 2 | 18 | 70 | 7 | 0 |
| Erigo | 2 | 3 | 13 | 70 | 5 | 1 |
| Erigo | 2 | 4 | 20 | 70 | 14 | 0 |
| Erigo | 2 | 5 | 21 | 70 | n.a. | 0 |
| Erigo | 2 | 6 | 22 | 70 | 11 | 0 |
| Tilt table | 4 | 1 | 20 | 50 | 0 | 0 |
| Tilt table | 4 | 2 | 10 | 45 | 0 | 0 |
| Tilt table | 4 | 3 | 25 | 70 | 10 | 0 |
| Tilt table | 4 | 4 | 15 | 70 | 5 | 1 |
| Tilt table | 4 | 5 | 20 | 50 | 0 | 1 |
| Tilt table | 4 | 6 | 22 | 70 | 10 | 0 |
| Tilt table | 4 | 7 | 30 | 70 | 13 | 0 |
| Tilt table | 4 | 8 | 17 | 70 | 2 | 1 |
| Tilt table | 4 | 9 | 15 | 60 | 0 | 1 |
| Tilt table | 4 | 10 | 10 | 70 | 2 | 1 |
| Tilt table | 5 | 1 | 45 | 70 | 5 | 0 |
| Tilt table | 5 | 2 | 28 | 70 | 5 | 1 |
| Tilt table | 5 | 3 | 33 | 70 | 7 | 0 |
| Tilt table | 5 | 4 | 32 | 70 | 13 | 0 |
| Tilt table | 5 | 5 | 15 | 60 | 0 | 1 |
| Tilt table | 5 | 6 | 25 | 70 | 8 | 1 |
| Tilt table | 5 | 7 | 30 | 70 | 7 | 0 |
| Tilt table | 5 | 8 | 22 | 70 | 8 | 0 |
| Tilt table | 5 | 9 | 22 | 70 | 7 | 0 |
| Tilt table | 5 | 10 | 27 | 70 | 9 | 0 |
| Tilt table | 6 | 1 | 30 | 70 | 15 | 1 |
| Tilt table | 6 | 2 | 12 | 50 | 0 | 1 |
| Erigo | 7 | 1 | 30 | 70 | 19 | 0 |
| Erigo | 7 | 2 | 25 | 70 | 13 | 0 |
| Erigo | 7 | 3 | 35 | 70 | 15 | 0 |
| Erigo | 7 | 4 | 25 | 70 | 10 | 0 |
| Erigo | 7 | 5 | 37 | 70 | 23 | 0 |
| Erigo | 7 | 6 | 30 | 70 | 10 | 0 |
| Erigo | 7 | 7 | 45 | 65 | 0 | 0 |
| Erigo | 7 | 8 | 32 | 70 | 20 | 0 |
| Erigo | 7 | 9 | 35 | 70 | 20 | 0 |
| Erigo | 7 | 10 | 30 | 75 | 10 | 0 |
| Erigo | 8 | 1 | 21 | 70 | 3 | 0 |
| Erigo | 8 | 2 | 23 | 70 | 3 | 0 |
| Erigo | 8 | 3 | 9 | 30 | 0 | 1 |
| Erigo | 8 | 4 | 18 | 70 | 4 | 0 |
| Erigo | 8 | 5 | 18 | 70 | 5 | 0 |
| Erigo | 8 | 6 | 23 | 70 | 6 | 0 |
| Erigo | 8 | 7 | 20 | 50 | 0 | 1 |
| Erigo | 8 | 8 | 17 | 70 | 2 | 0 |
| Erigo | 8 | 9 | 10 | 70 | 2 | 1 |
| Erigo | 8 | 10 | 25 | 50 | 0 | 1 |
| Erigo | 9 | 1 | 24 | 70 | 7 | 0 |
| Erigo | 9 | 2 | 27 | 70 | 7 | 0 |
| Erigo | 9 | 3 | 35 | 70 | 20 | 0 |
| Erigo | 9 | 4 | 24 | 70 | 12 | 0 |
| Erigo | 9 | 5 | 24 | 70 | 13 | 0 |
| Erigo | 9 | 6 | 27 | 70 | 14 | 0 |
| Erigo | 9 | 7 | 25 | 70 | 6 | 0 |
| Erigo | 9 | 8 | 32 | 70 | 9 | 0 |
| Erigo | 9 | 9 | 25 | 75 | 15 | 0 |
| Erigo | 9 | 10 | 25 | 70 | 12 | 0 |
| Tilt table | 10 | 1 | 30 | 70 | 15 | 0 |
| Tilt table | 10 | 2 | 40 | 70 | 22 | 0 |
| Tilt table | 10 | 3 | 40 | 70 | 15 | 0 |
| Tilt table | 10 | 4 | 40 | 70 | 20 | 0 |
| Tilt table | 10 | 5 | 35 | 60 | 0 | 0 |
| Tilt table | 10 | 6 | 28 | 70 | 13 | 0 |
| Tilt table | 10 | 7 | 35 | 70 | 10 | 0 |
| Tilt table | 10 | 8 | 46 | 70 | 25 | 0 |
| Tilt table | 10 | 9 | 23 | 70 | 10 | 0 |
| Tilt table | 10 | 10 | 25 | 70 | 15 | 0 |
| Tilt table | 12 | 1 | 17 | 70 | 5 | 1 |
| Tilt table | 12 | 2 | 23 | 70 | 6 | 1 |
| Tilt table | 12 | 3 | 15 | 70 | 5 | 1 |
| Tilt table | 12 | 4 | 17 | 70 | 10 | 0 |
| Tilt table | 12 | 5 | 23 | 70 | 9 | 0 |
| Tilt table | 12 | 6 | 22 | 70 | 14 | 1 |
| Tilt table | 12 | 7 | 19 | 70 | 4 | 0 |
| Tilt table | 12 | 8 | 14 | 70 | 9 | 1 |
| Tilt table | 12 | 9 | 17 | 70 | 10 | 0 |
| Tilt table | 12 | 10 | 19 | 70 | 13 | 0 |
| Tilt table | 13 | 1 | 25 | 70 | 5 | 0 |
| Tilt table | 13 | 2 | 18 | 70 | 2 | 1 |
| Tilt table | 13 | 3 | 20 | 30 | 0 | 1 |
| Tilt table | 13 | 4 | 14 | 30 | 0 | 1 |
| Tilt table | 13 | 5 | 26 | 70 | 4 | 1 |
| Tilt table | 13 | 6 | 37 | 20 | 0 | 1 |
| Tilt table | 13 | 7 | 27 | 60 | 0 | 0 |
| Tilt table | 13 | 8 | 24 | 70 | 5 | 1 |
| Tilt table | 13 | 9 | 29 | 70 | 6 | 0 |
| Tilt table | 13 | 10 | 28 | 60 | 0 | 0 |
| Tilt table | 14 | 1 | 12 | 70 | 5 | 1 |
| Tilt table | 14 | 2 | 24 | 50 | 0 | 1 |
| Tilt table | 14 | 3 | 15 | 50 | 0 | 1 |
| Tilt table | 14 | 4 | 24 | 50 | 0 | 1 |
| Tilt table | 14 | 5 | 30 | 70 | 6 | 1 |
| Tilt table | 14 | 6 | 24 | 70 | 5 | 0 |
| Tilt table | 14 | 7 | 26 | 70 | 6 | 0 |
| Tilt table | 14 | 8 | 25 | 70 | 5 | 0 |
| Tilt table | 14 | 9 | 23 | 70 | 5 | 1 |
| Tilt table | 14 | 10 | 30 | 70 | 8 | 1 |
| Erigo | 16 | 1 | 25 | 70 | 10 | 0 |
| Erigo | 16 | 2 | 25 | 70 | 18 | 0 |
| Erigo | 16 | 3 | 26 | 70 | 17 | 0 |
| Erigo | 16 | 4 | 24 | 70 | 16 | 1 |
| Erigo | 16 | 5 | 29 | 70 | 22 | 0 |
| Erigo | 16 | 6 | 30 | 70 | 20 | 0 |
| Erigo | 16 | 7 | 21 | 70 | 14 | 0 |
| Erigo | 16 | 8 | 23 | 70 | 7 | 0 |
| Erigo | 16 | 9 | 24 | 70 | 5 | 0 |
| Erigo | 16 | 10 | 35 | 70 | 17 | 1 |
| Erigo | 17 | 1 | 20 | 70 | 9 | 1 |
| Erigo | 17 | 2 | 25 | 70 | 11 | 0 |
| Erigo | 17 | 3 | 31 | 70 | 15 | 0 |
| Erigo | 17 | 4 | 23 | 70 | 8 | 0 |
| Erigo | 17 | 5 | 25 | 70 | 13 | 0 |
| Erigo | 17 | 6 | 45 | 70 | 15 | 0 |
| Erigo | 17 | 7 | 35 | 70 | 5 | 0 |
| Erigo | 17 | 8 | 17 | 70 | 3 | 1 |
| Erigo | 17 | 9 | 25 | 70 | 2 | 0 |
| Erigo | 17 | 10 | 24 | 70 | 3 | 1 |
| Erigo | 18 | 1 | 15 | 50 | 0 | 0 |
| Erigo | 18 | 2 | 28 | 50 | 0 | 0 |
| Erigo | 18 | 3 | 21 | 60 | 0 | 0 |
| Erigo | 18 | 4 | 20 | 60 | 0 | 0 |
| Erigo | 18 | 5 | 23 | 65 | 0 | 0 |
| Erigo | 18 | 6 | 22 | 60 | 0 | 0 |
| Erigo | 18 | 7 | 23 | 65 | 0 | 0 |
| Erigo | 18 | 8 | 23 | 70 | 15 | 0 |
| Erigo | 18 | 9 | 25 | 70 | 16 | 0 |
| Erigo | 18 | 10 | 27 | 70 | 19 | 0 |
| Tilt table | 19 | 1 | 15 | 70 | 10 | 1 |
| Tilt table | 19 | 2 | 10 | 70 | 6 | 1 |
| Tilt table | 19 | 3 | 25 | 70 | 22 | 0 |
| Tilt table | 19 | 4 | 22 | 70 | 15 | 1 |
| Tilt table | 19 | 5 | 32 | 70 | 16 | 0 |
| Tilt table | 19 | 6 | 22 | 70 | 7 | 0 |
| Tilt table | 19 | 7 | 23 | 70 | 7 | 0 |
| Tilt table | 19 | 8 | 25 | 70 | 8 | 0 |
| Tilt table | 19 | 9 | 31 | 70 | 9 | 0 |
| Tilt table | 19 | 10 | 35 | 70 | 10 | 0 |
| Tilt table | 20 | 1 | 25 | 50 | 0 | 1 |
| Tilt table | 20 | 2 | 4 | 30 | 0 | 1 |
| Tilt table | 20 | 3 | 23 | 50 | 0 | 1 |
| Tilt table | 20 | 4 | 15 | 30 | 0 | 1 |
| Tilt table | 20 | 5 | 13 | 50 | 0 | 1 |
| Tilt table | 20 | 6 | 16 | 50 | 0 | 1 |
| Tilt table | 20 | 7 | 10 | 30 | 0 | 1 |
| Tilt table | 20 | 8 | 13 | 70 | 2 | 1 |
| Tilt table | 20 | 9 | 16 | 30 | 0 | 1 |
| Tilt table | 20 | 10 | 12 | 50 | 0 | 1 |
| Erigo | 21 | 1 | 19 | 70 | 7 | 1 |
| Erigo | 21 | 2 | 20 | 70 | 20 | 1 |
| Erigo | 21 | 3 | 30 | 70 | 10 | 0 |
| Erigo | 21 | 4 | 23 | 70 | 10 | 1 |
| Erigo | 21 | 5 | 26 | 70 | 17 | 0 |
| Erigo | 21 | 6 | 32 | 70 | 18 | 0 |
| Erigo | 21 | 7 | 24 | 70 | 14 | 1 |
| Erigo | 21 | 8 | 26 | 70 | 9 | 1 |
| Erigo | 21 | 9 | 30 | 70 | 19 | 0 |
| Erigo | 21 | 10 | 28 | 70 | 16 | 0 |
| Erigo | 22 | 1 | 29 | 70 | 16 | 1 |
| Erigo | 22 | 2 | 31 | 70 | 15 | 0 |
| Erigo | 22 | 3 | 23 | 70 | 13 | 0 |
| Erigo | 22 | 4 | 21 | 70 | 14 | 0 |
| Erigo | 22 | 5 | 23 | 70 | 13 | 0 |
| Erigo | 22 | 6 | 25 | 70 | 19 | 0 |
| Erigo | 23 | 1 | 25 | 70 | 4 | 0 |
| Erigo | 23 | 2 | 19 | 70 | 7 | 0 |
| Erigo | 23 | 3 | 33 | 70 | 19 | 0 |
| Erigo | 23 | 4 | 29 | 70 | 13 | 0 |
| Erigo | 23 | 5 | 23 | 70 | 15 | 0 |
| Erigo | 23 | 6 | 31 | 70 | 25 | 0 |
| Erigo | 23 | 7 | 31 | 70 | 22 | 0 |
| Erigo | 23 | 8 | 30 | 70 | 24 | 0 |
| Erigo | 23 | 9 | 29 | 70 | 24 | 0 |
| Erigo | 23 | 10 | 29 | 70 | 24 | 0 |
| Tilt table | 25 | 1 | 30 | 70 | 24 | 1 |
| Tilt table | 25 | 2 | 28 | 70 | 18 | 0 |
| Tilt table | 25 | 3 | 13 | 70 | 5 | 1 |
| Tilt table | 25 | 4 | 31 | 70 | 21 | 0 |
| Tilt table | 25 | 5 | 22 | 70 | 11 | 1 |
| Tilt table | 25 | 6 | 38 | 70 | 22 | 0 |
| Tilt table | 25 | 7 | 40 | 70 | 27 | 0 |
| Tilt table | 25 | 8 | 32 | 70 | 27 | 0 |
| Tilt table | 25 | 9 | 25 | 70 | 21 | 0 |
| Tilt table | 25 | 10 | 27 | 70 | 21 | 0 |
| Tilt table | 26 | 1 | 24 | 70 | 6 | 0 |
| Tilt table | 26 | 2 | 13 | 70 | 5 | 1 |
| Tilt table | 26 | 3 | 19 | 70 | 3 | 1 |
| Tilt table | 26 | 4 | 9 | 70 | 2 | 1 |
| Tilt table | 26 | 5 | 10 | 70 | 4 | 1 |
| Tilt table | 26 | 6 | 16 | 70 | 4 | 1 |
| Tilt table | 26 | 7 | 20 | 70 | 9 | 1 |
| Tilt table | 26 | 8 | 15 | 70 | 8 | 1 |
| Tilt table | 26 | 9 | 20 | 70 | 11 | 0 |
| Tilt table | 26 | 10 | 16 | 70 | 10 | 1 |
| Erigo | 27 | 1 | 24 | 70 | 10 | 0 |
| Erigo | 27 | 2 | 24 | 70 | 4 | 0 |
| Erigo | 27 | 3 | 22 | 70 | 17 | 0 |
| Erigo | 27 | 4 | 20 | 70 | 10 | 0 |
| Erigo | 27 | 5 | 23 | 70 | 20 | 0 |
| Erigo | 27 | 6 | 26 | 70 | 20 | 0 |
| Erigo | 27 | 7 | 25 | 70 | 20 | 0 |
| Erigo | 27 | 8 | 24 | 70 | 20 | 0 |
| Erigo | 27 | 9 | 23 | 70 | 22 | 0 |
| Erigo | 27 | 10 | 11 | 70 | 7 | 1 |
| Tilt table | 28 | 1 | 12 | 50 | 0 | 1 |
| Tilt table | 28 | 2 | 12 | 50 | 0 | 1 |
| Tilt table | 28 | 3 | 8 | 50 | 0 | 1 |
| Tilt table | 28 | 4 | 3 | 30 | 0 | 1 |
| Tilt table | 28 | 5 | 22 | 70 | 11 | 0 |
| Tilt table | 28 | 6 | 5 | 30 | 0 | 1 |
| Tilt table | 28 | 7 | 28 | 70 | 20 | 0 |
| Tilt table | 28 | 8 | 30 | 70 | 23 | 1 |
| Tilt table | 28 | 9 | 25 | 70 | 16 | 1 |
| Tilt table | 28 | 10 | 8 | 50 | 0 | 1 |
| Tilt table | 29 | 1 | 30 | 70 | 19 | 0 |
| Tilt table | 29 | 2 | 35 | 70 | 10 | 0 |
| Tilt table | 29 | 3 | 40 | 70 | 15 | 0 |
| Tilt table | 29 | 4 | 23 | 80 | 5 | 1 |
| Tilt table | 29 | 5 | 20 | 30 | 0 | 1 |
| Tilt table | 29 | 6 | 23 | 60 | 0 | 0 |
| Tilt table | 29 | 7 | 27 | 50 | 0 | 0 |
| Tilt table | 29 | 8 | 23 | 50 | 0 | 0 |
| Tilt table | 29 | 9 | 25 | 60 | 0 | 1 |
| Tilt table | 29 | 10 | 25 | 40 | 0 | 0 |
| Erigo | 30 | 1 | 33 | 70 | 5 | 0 |
| Erigo | 30 | 2 | 34 | 70 | 5 | 0 |
| Erigo | 30 | 3 | 32 | 70 | 9 | 0 |
| Erigo | 30 | 4 | 33 | 70 | 7 | 0 |
| Erigo | 30 | 5 | 29 | 70 | 11 | 0 |
| Erigo | 30 | 6 | 30 | 70 | 15 | 0 |
| Erigo | 30 | 7 | 32 | 70 | 12 | 0 |
| Erigo | 30 | 8 | 26 | 70 | 5 | 0 |
| Erigo | 30 | 9 | 30 | 70 | 13 | 0 |
| Erigo | 30 | 10 | 33 | 70 | 13 | 0 |
| Tilt table | 31 | 1 | 35 | 70 | 14 | 0 |
| Tilt table | 31 | 2 | 28 | 70 | 5 | 0 |
| Tilt table | 31 | 3 | 31 | 70 | 15 | 0 |
| Tilt table | 31 | 4 | 20 | 85 | 5 | 0 |
| Tilt table | 31 | 5 | 20 | 80 | 5 | 0 |
| Tilt table | 31 | 6 | 25 | 80 | 15 | 0 |
| Tilt table | 31 | 7 | 24 | 80 | 10 | 0 |
| Tilt table | 31 | 8 | 27 | 80 | 10 | 0 |
| Tilt table | 31 | 9 | 28 | 80 | 10 | 0 |
| Tilt table | 31 | 10 | 29 | 80 | 11 | 0 |
| Erigo | 32 | 1 | 19 | 70 | 4 | 0 |
| Erigo | 32 | 2 | 24 | 70 | 11 | 0 |
| Erigo | 32 | 3 | 21 | 70 | 14 | 0 |
| Erigo | 32 | 4 | 21 | 70 | 9 | 0 |
| Erigo | 32 | 5 | 26 | 70 | 13 | 0 |
| Erigo | 32 | 6 | 25 | 70 | 15 | 0 |
| Erigo | 32 | 7 | 27 | 70 | 14 | 0 |
| Erigo | 32 | 8 | 28 | 70 | 8 | 0 |
| Erigo | 32 | 9 | 23 | 70 | 9 | 0 |
| Erigo | 32 | 10 | 26 | 70 | 12 | 0 |
| Tilt table | 33 | 1 | 26 | 40 | 0 | 0 |
| Tilt table | 33 | 2 | 30 | 40 | 0 | 0 |
| Tilt table | 33 | 3 | 30 | 40 | 0 | 0 |
| Tilt table | 33 | 4 | 21 | 70 | 5 | 1 |
| Tilt table | 33 | 5 | 31 | 70 | 9 | 0 |
| Tilt table | 33 | 6 | 32 | 50 | 0 | 0 |
| Tilt table | 33 | 7 | 32 | 50 | 0 | 0 |
| Tilt table | 33 | 8 | 27 | 60 | 0 | 1 |
| Tilt table | 33 | 9 | n.a. | n.a. | n.a. | n.a. |
| Tilt table | 33 | 10 | n.a. | n.a. | n.a. | n.a. |
| Erigo | 34 | 1 | 20 | 50 | 0 | 1 |
| Erigo | 34 | 2 | 21 | 70 | 2 | 1 |
| Erigo | 34 | 3 | 22 | 70 | 8 | 0 |
| Erigo | 34 | 4 | 21 | 70 | 6 | 0 |
| Erigo | 34 | 5 | 24 | 70 | 11 | 0 |
| Erigo | 34 | 6 | 21 | 70 | 6 | 0 |
| Erigo | 34 | 7 | 24 | 70 | 6 | 0 |
| Erigo | 34 | 8 | 29 | 70 | 8 | 0 |
| Erigo | 34 | 9 | 25 | 70 | 7 | 0 |
| Erigo | 34 | 10 | 25 | 70 | 8 | 0 |
| Erigo | 36 | 1 | 28 | 55 | 0 | 0 |
| Erigo | 36 | 2 | 21 | 55 | 0 | 0 |
| Erigo | 36 | 3 | 26 | 60 | 0 | 0 |
| Erigo | 36 | 4 | 30 | 55 | 0 | 0 |
| Erigo | 36 | 5 | 26 | 55 | 0 | 0 |
| Erigo | 36 | 6 | 23 | 75 | 1 | 1 |
| Erigo | 36 | 7 | 23 | 70 | 3 | 1 |
| Erigo | 36 | 8 | 22 | 75 | 3 | 1 |
| Erigo | 36 | 9 | 22 | 70 | 10 | 0 |
| Erigo | 36 | 10 | 33 | 75 | 18 | 0 |
| Tilt table | 37 | 1 | 13 | 30 | 0 | 1 |
| Tilt table | 37 | 2 | 29 | 70 | 8 | 0 |
| Tilt table | 37 | 3 | 23 | 70 | 4 | 0 |
| Tilt table | 37 | 4 | 21 | 70 | 12 | 0 |
| Tilt table | 37 | 5 | 28 | 70 | 21 | 0 |
| Tilt table | 37 | 6 | 26 | 70 | 10 | 0 |
| Tilt table | 37 | 7 | 26 | 70 | 20 | 0 |
| Tilt table | 37 | 8 | 20 | 70 | 12 | 0 |
| Tilt table | 37 | 9 | 16 | 70 | 11 | 1 |
| Tilt table | 37 | 10 | 20 | 70 | 12 | 0 |
| Tilt table | 38 | 1 | 25 | 70 | 4 | 1 |
| Tilt table | 38 | 2 | 31 | 70 | 8 | 1 |
| Tilt table | 38 | 3 | 0 | 0 | 0 | 1 |
| Tilt table | 38 | 4 | 19 | 70 | 3 | 1 |
| Tilt table | 38 | 5 | 25 | 70 | 6 | 1 |
| Tilt table | 38 | 6 | 0 | 0 | 0 | 1 |
| Tilt table | 38 | 7 | 20 | 70 | 1 | 1 |
| Tilt table | 38 | 8 | n.a. | n.a. | n.a. | n.a. |
| Tilt table | 38 | 9 | n.a. | n.a. | n.a. | n.a. |
| Tilt table | 38 | 10 | n.a. | n.a. | n.a. | n.a. |
| Tilt table | 39 | 1 | 16 | 60 | 0 | 0 |
| Tilt table | 39 | 2 | 24 | 75 | 3 | 0 |
| Tilt table | 39 | 3 | 23 | 75 | 10 | 0 |
| Tilt table | 39 | 4 | 20 | 80 | 4 | 1 |
| Tilt table | 39 | 5 | 19 | 75 | 2 | 0 |
| Tilt table | 39 | 6 | 18 | 70 | 7 | 0 |
| Tilt table | 39 | 7 | 23 | 70 | 5 | 0 |
| Tilt table | 39 | 8 | 23 | 60 | 0 | 0 |
| Tilt table | 39 | 9 | 24 | 75 | 6 | 0 |
| Tilt table | 39 | 10 | 31 | 75 | 5 | 0 |
| Erigo | 40 | 1 | 26 | 80 | 3 | 0 |
| Erigo | 40 | 2 | 30 | 80 | 9 | 0 |
| Erigo | 40 | 3 | 27 | 80 | 4 | 0 |
| Erigo | 40 | 4 | 24 | 70 | 5 | 0 |
| Erigo | 40 | 5 | 23 | 70 | 9 | 0 |
| Erigo | 40 | 6 | 27 | 70 | 17 | 0 |
| Erigo | 40 | 7 | 26 | 70 | 13 | 0 |
| Erigo | 40 | 8 | 31 | 70 | 13 | 0 |
| Erigo | 40 | 9 | 31 | 70 | 10 | 0 |
| Erigo | 40 | 10 | 37 | 83 | 8 | 0 |
| Erigo | 41 | 1 | 25 | 70 | 8 | 0 |
| Erigo | 41 | 2 | 24 | 70 | 14 | 0 |
| Erigo | 41 | 3 | 25 | 70 | 16 | 0 |
| Erigo | 41 | 4 | 28 | 70 | 13 | 0 |
| Erigo | 41 | 5 | 25 | 70 | 16 | 0 |
| Erigo | 41 | 6 | 25 | 70 | 17 | 0 |
| Erigo | 41 | 7 | 25 | 70 | 17 | 0 |
| Erigo | 41 | 8 | 24 | 70 | 17 | 0 |
| Erigo | 41 | 9 | 30 | 70 | 18 | 0 |
| Erigo | 41 | 10 | 29 | 70 | 16 | 0 |
| Erigo | 42 | 1 | 21 | 70 | 8 | 0 |
| Erigo | 42 | 2 | 32 | 70 | 27 | 0 |
| Erigo | 42 | 3 | 22 | 70 | 13 | 0 |
| Erigo | 42 | 4 | 25 | 70 | 15 | 0 |
| Erigo | 42 | 5 | 19 | 70 | 10 | 0 |
| Erigo | 42 | 6 | 27 | 70 | 15 | 0 |
| Erigo | 42 | 7 | 25 | 70 | 17 | 0 |
| Erigo | 42 | 8 | 29 | 70 | 18 | 0 |
| Erigo | 42 | 9 | n.a. | n.a. | n.a. | n.a. |
| Erigo | 42 | 10 | n.a. | n.a. | n.a. | n.a. |
| Tilt table | 43 | 1 | 15 | 30 | 0 | 1 |
| Tilt table | 43 | 2 | 30 | 50 | 0 | 0 |
| Tilt table | 43 | 3 | 25 | 50 | 0 | 1 |
| Tilt table | 43 | 4 | 27 | 50 | 0 | 0 |
| Tilt table | 43 | 5 | 25 | 50 | 0 | 1 |
| Tilt table | 43 | 6 | 35 | 70 | 7 | 0 |
| Tilt table | 43 | 7 | n.a. | n.a. | n.a. | n.a. |
| Tilt table | 43 | 8 | 20 | 70 | 5 | 0 |
| Tilt table | 43 | 9 | 30 | 40 | 0 | 0 |
| Tilt table | 43 | 10 | 31 | 40 | 0 | 0 |
| Erigo | 44 | 1 | 21 | 30 | 0 | 0 |
| Erigo | 44 | 2 | 28 | 70 | 4 | 0 |
| Erigo | 44 | 3 | 20 | 70 | 17 | 0 |
| Erigo | 44 | 4 | 25 | 70 | 2 | 0 |
| Erigo | 44 | 5 | 25 | 70 | 11 | 0 |
| Erigo | 44 | 6 | 24 | 70 | 5 | 0 |
| Erigo | 44 | 7 | 23 | 70 | 7 | 0 |
| Erigo | 44 | 8 | 28 | 70 | 10 | 0 |
| Erigo | 44 | 9 | 23 | 70 | 10 | 0 |
| Erigo | 44 | 10 | n.a. | n.a. | n.a. | n.a. |
| Tilt table | 45 | 1 | 24 | 50 | 0 | 0 |
| Tilt table | 45 | 2 | 22 | 50 | 0 | 0 |
| Tilt table | 45 | 3 | 21 | 70 | 7 | 0 |
| Tilt table | 45 | 4 | 26 | 50 | 0 | 0 |
| Tilt table | 45 | 5 | 17 | 50 | 0 | 0 |
| Tilt table | 45 | 6 | 24 | 70 | 5 | 0 |
| Tilt table | 45 | 7 | 15 | 70 | 3 | 1 |
| Tilt table | 45 | 8 | 15 | 50 | 0 | 1 |
| Tilt table | 45 | 9 | 17 | 50 | 0 | 1 |
| Tilt table | 45 | 10 | n.a. | n.a. | n.a. | n.a. |
| Tilt table | 47 | 1 | 30 | 70 | 10 | 1 |
| Tilt table | 47 | 2 | 25 | 70 | 3 | 1 |
| Tilt table | 47 | 3 | 20 | 70 | 19 | 0 |
| Tilt table | 47 | 4 | 37 | 70 | 31 | 0 |
| Tilt table | 47 | 5 | 29 | 70 | 7 | 1 |
| Tilt table | 47 | 6 | 31 | 70 | 29 | 1 |
| Tilt table | 47 | 7 | 22 | 80 | 15 | 0 |
| Tilt table | 47 | 8 | 36 | 70 | 7 | 1 |
| Tilt table | 47 | 9 | 32 | 70 | 22 | 0 |
| Tilt table | 47 | 10 | 38 | 70 | 30 | 0 |
| Tilt table | 49 | 1 | 17 | 70 | 5 | 1 |
| Tilt table | 49 | 2 | 20 | 70 | 5 | 1 |
| Tilt table | 49 | 3 | 17 | 70 | 3 | 1 |
| Tilt table | 49 | 4 | 22 | 50 | 0 | 1 |
| Tilt table | 49 | 5 | 14 | 50 | 0 | 1 |
| Tilt table | 49 | 6 | 14 | 50 | 0 | 1 |
| Tilt table | 49 | 7 | 19 | 50 | 0 | 1 |
| Tilt table | 49 | 8 | n.a. | n.a. | n.a. | n.a. |
| Erigo | 50 | 1 | 10 | 0 | 0 | 1 |
| Erigo | 50 | 2 | 18 | 70 | 5 | 1 |
| Erigo | 50 | 3 | n.a. | n.a. | n.a. | n.a. |

Abbr.: n.a., no therapy applied or not documented; interruption 0, no therapy interruption; interruption 1, therapy interruption necessary.
